# The Bub1–Plk1 kinase complex promotes spindle checkpoint signalling through Cdc20 phosphorylation

**DOI:** 10.1038/ncomms10818

**Published:** 2016-02-25

**Authors:** Luying Jia, Bing Li, Hongtao Yu

**Affiliations:** 1 Department of Pharmacology, Howard Hughes Medical Institute, University of Texas Southwestern Medical Center, 6001 Forest Park Road, Dallas, Texas 75390, USA

## Abstract

The spindle checkpoint senses unattached kinetochores and inhibits the Cdc20-bound anaphase-promoting complex or cyclosome (APC/C), to delay anaphase, thereby preventing aneuploidy. A critical checkpoint inhibitor of APC/C^Cdc20^ is the mitotic checkpoint complex (MCC). It is unclear whether MCC suffices to inhibit all cellular APC/C. Here we show that human checkpoint kinase Bub1 not only directly phosphorylates Cdc20, but also scaffolds Plk1-mediated phosphorylation of Cdc20. Phosphorylation of Cdc20 by Bub1–Plk1 inhibits APC/C^Cdc20^ *in vitro* and is required for checkpoint signalling in human cells. Bub1–Plk1-dependent Cdc20 phosphorylation is regulated by upstream checkpoint signals and is dispensable for MCC assembly. A phospho-mimicking Cdc20 mutant restores nocodazole-induced mitotic arrest in cells depleted of Mad2 or BubR1. Thus, Bub1–Plk1-mediated phosphorylation of Cdc20 constitutes an APC/C-inhibitory mechanism that is parallel, but not redundant, to MCC formation. Both mechanisms are required to sustain mitotic arrest in response to spindle defects.

## Abstract (web summary)

The mitotic checkpoint complex (MCC) inhibits the anaphase-promoting complex (APC/C) bound to Cdc20 in response to spindle defects. Here, the authors show that Bub1-Plk1-mediated phosphorylation of Cdc20 constitutes a parallel, non-redundant APC/C-inhibitory mechanism in addition to MCC activity.

## Graphical abstract

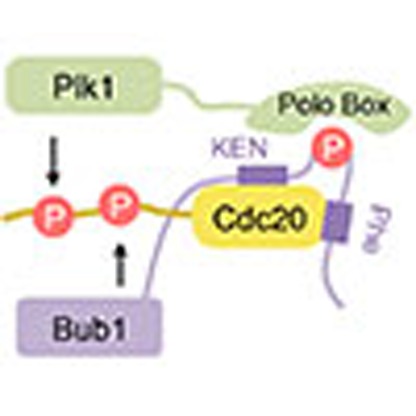

The spindle checkpoint ensures the fidelity of chromosome segregation^1^,^2^,^3^. Chromosome missegregation during mitosis can result in aneuploidy, which can promote tumorigenesis depending on context. Unattached kinetochores recruit and activate checkpoint proteins to produce diffusible anaphase inhibitors, which inhibit the ubiquitin ligase activity of the anaphase-promoting complex/cyclosome (APC/C) bound to Cdc20 (refs 4, 5). Inhibition of APC/C^Cdc20^ stabilizes securin and cyclin B1, and delays sister chromatid separation and exit from mitosis. Proper microtubule attachment to kinetochores releases the checkpoint proteins and turns off the checkpoint^6^,^7^,^8^. APC/C^Cdc20^ ubiquitinates securin and cyclin B1 to trigger their degradation, promoting the onset of anaphase.

Cdc20 activates APC/C in part through directly contributing to binding of APC/C degrons found in substrates, including the destruction (D) box, the KEN box and the recently discovered Phe box (also called ABBA motif)^9^,^10^,^11^,^12^,^13^,^14^,^15^. BubR1 and Mad2 can each independently inhibit APC/C^Cdc20^ using different mechanisms *in vitro*^15^,^16^,^17^ and can collaborate to inhibit APC/C^Cdc20^ *in vivo* by forming the mitotic checkpoint complex (MCC) that consists of the constitutive BubR1–Bub3 complex, Cdc20 and Mad2 (refs 18, 19). Unattached kinetochores promote the conformational activation of Mad2, which enables Mad2 binding to Cdc20 (refs 20, 21). The Mad2–Cdc20 complex then associates with BubR1–Bub3 at kinetochores to form MCC^22^. MCC blocks substrate recruitment by APC/C^Cdc20^ in two ways: anchoring Cdc20 to a binding site on APC/C incompatible for substrate ubiquitination and acting as a competitive inhibitor of substrate recruitment through D and KEN boxes of BubR1 (refs 12, 15, 19, 23, 24, 25, 26).

Kinetochore-enhanced MCC production is clearly required for APC/C^Cdc20^ inhibition during checkpoint signalling^1^,^2^,^3^. It is less clear whether MCC as a stoichiometric inhibitor is sufficient to inhibit all cellular APC/C. We have previously shown that the checkpoint kinase Bub1 directly phosphorylates Cdc20 and inhibits APC/C^Cdc20^, implicating the existence of other APC/C inhibitory mechanisms^27^. On the other hand, the kinase activity of Bub1 is not strictly required for the spindle checkpoint in human cells^28^,^29^. Furthermore, in the mouse, the checkpoint functions of the Bub1 kinase activity have been attributed to mechanisms aside from Cdc20 phosphorylation^30^. The functional relevance of Bub1-dependent Cdc20 phosphorylation needs to be further clarified.

Plk1 is a cell cycle kinase with myriad functions, including spindle assembly and chromosome alignment^31^. Both Bub1 and BubR1 contain an STP motif that, when phosphorylated by Cdk1 in mitosis, binds to the polo-box domain of Plk1 (refs 32, 33). Plk1 phosphorylates the KARD motif of BubR1 to enable PP2A binding^34^. BubR1–Plk1-dependent recruitment of PP2A to kinetochores promotes chromosome alignment at metaphase^34^. The Bub1–Plk1 interaction recruits a population of Plk1 to kinetochores^32^, but the functional substrate of Bub1–Plk1 at kinetochores remains to be identified.

Here we show that in addition to directly phosphorylating Cdc20, the non-kinase domains of Bub1 bind to both Plk1 and Cdc20, thus providing a scaffold for Cdc20 phosphorylation by Plk1. Phosphorylation of Cdc20 by the Bub1–Plk1 complex inhibits APC/C^Cdc20^ *in vitro* and is required for and regulated by checkpoint signalling in human cells, but is dispensable for MCC formation. A Cdc20 mutant mimicking a major Plk1 phosphorylation event rescues the checkpoint defects of cells depleted of Mad2 or BubR1. Our study extends the scaffolding roles of the checkpoint kinase Bub1 and establishes Cdc20 phosphorylation by Bub1–Plk1 as a critical mechanism that acts in parallel to MCC formation, to inhibit APC/C^Cdc20^.

## Results

### Human Plk1 and Bub1 cooperate in the spindle checkpoint

**Figure 1:**
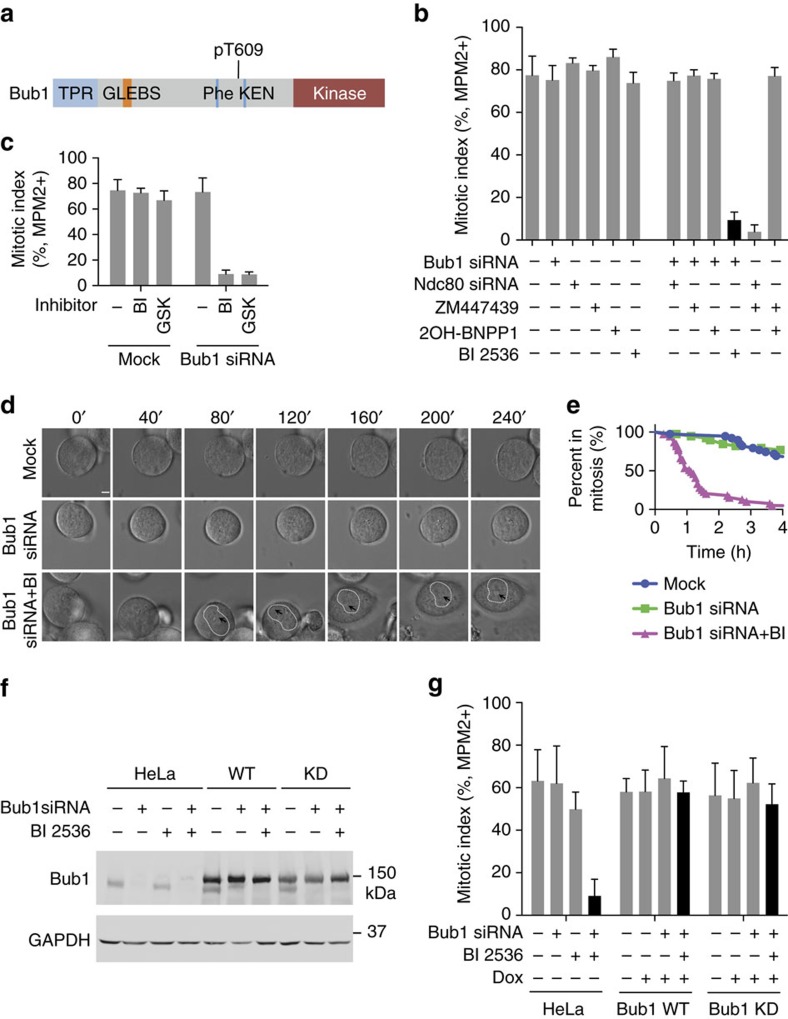
Bub1 depletion and Plk1 inhibition synergize to inactivate the spindle checkpoint. (**a**) Domains and motifs of Bub1. GLEBS, Gle2-binding sequence; TPR, tetratricopeptide repeat. (**b**) Quantification of the mitotic index (defined as the percentage of MPM2+, 4N cells) of HeLa Tet-On cells treated with 5 μM nocodazole and the indicated siRNAs and kinase inhibitors. Mean±s.d. for columns 1, 2, 5, 6 and 10 (3 or more independent experiments); mean±range for other columns (2 independent experiments). (**c**) Quantification of the mitotic index of HeLa Tet-On cells treated with 500 nM nocodazole and the indicated siRNA and Plk1 inhibitors (BI, BI 2536; GSK, GSK461364). Mean±s.d. (*n*=3 independent experiments). (**d**) Differential interference contrast (DIC) images of representative cells with the indicated siRNA and inhibitor treatments at different time points in 200 nM taxol. Time 0 marks the start of imaging and is ∼40 min after the addition of BI 2536. Dashed lines demarcate nucleus. Arrowheads indicate nucleoli. Scale bar, 5 μm. (**e**) Quantification of the percentage of cells in **d** remaining in mitosis at different time points (*n*=39 cells for each group). (**f**) Immunoblots of lysates of parental HeLa Tet-On cells and cells stably expressing GFP-Bub1 WT or kinase-dead mutant (KD) treated with doxycycline in the presence or absence of Bub1 siRNA and BI 2536. (**g**) Quantification of the mitotic index of HeLa Tet-On parental cells and cells expressing GFP-Bub1 WT and KD treated with 200 nM taxol in the presence or absence of doxycycline (Dox), Bub1 siRNA and BI 2536. Mean±s.d. (*n*=3 independent experiments).

Bub1 is a multifunctional component of the spindle checkpoint (Fig. 1a). However, it was difficult to produce checkpoint defects in human cells depleted of Bub1 with RNA interference (RNAi) (Supplementary Fig. 1a). Only Bub1 siRNA-d (initially reported by Meraldi and colleagues^29^) produced checkpoint defects. Multiple other Bub1 small interfering RNAs (siRNAs) failed to produce checkpoint defects, despite their ability to efficiently deplete Bub1 (Supplementary Fig. 1b,c). The checkpoint defects caused by Bub1 siRNA-d were rescued by an RNAi-resistant Bub1 transgene, indicating that the effects of this siRNA were Bub1 dependent^29^ (Supplementary Fig. 1d). Based on quantitative reverse transcriptase–PCR, Bub1 siRNA-d depleted Bub1 messenger RNA slightly more efficiently than siRNA-b and siRNA-c (Supplementary Fig. 1c). Thus, only siRNA-d might have depleted Bub1 below the threshold level required for checkpoint signalling. Alternatively, Bub1 siRNA-d might have depleted proteins other than Bub1 that cooperated with Bub1 in the checkpoint. Regardless, these results indicate that a small amount of Bub1 can sustain the spindle checkpoint.

Inactivation or partial depletion of several important mitotic regulators, including Aurora B, Ndc80 and Plk1, does not cause cells to escape from nocodazole-triggered mitotic arrest^35^,^36^,^37^,^38^, despite their known roles in regulating checkpoint proteins. We thus tested whether their inactivation synergized with Bub1 siRNA-b or siRNA-c, to override the mitotic arrest exerted by nocodazole. Consistent with previous studies^35^,^36^,^37^, inhibition of Aurora B with ZM44739 synergized with Ndc80 depletion to produce checkpoint defects in HeLa cells (Fig. 1b). Aurora B inhibition or Ndc80 depletion did not synergize with Bub1 depletion by siRNA-c to produce checkpoint defects. Addition of the Plk1 inhibitor BI 2536 to cells transfected with Bub1 siRNA-c and arrested in nocodazole caused them to undergo mitotic exit (Fig. 1b). As BI 2536 had targets other than Plk1 in human cells^39^, we tested another Plk1 inhibitor GSK461364 with this assay. GSK461364 also produced strong checkpoint defects in cells transfected with Bub1 siRNA-c (Fig. 1c). Without Bub1 depletion, BI 2536 or GSK461364 caused mitotic arrest in the absence of nocodazole (Supplementary Fig. 1e) and did not cause escape from nocodazole-mediated mitotic arrest (Fig. 1b,c). Bub1 depletion and BI 2536 treatment also caused HeLa cells to escape from taxol-induced mitotic arrest (Supplementary Fig. 1f).

We next used live-cell imaging to visualize this escape process (Fig. 1d). Mock-treated cells or cells treated with Bub1 siRNA alone remained arrested in mitosis during the 4-h imaging period (Fig. 1d,e). In contrast, about 50% of the cells treated with both Bub1 siRNA and BI 2536 escaped from mitosis within 1 h, as indicated by nucleus reformation.

BI 2536 also synergized with Bub1 siRNA-b to produce checkpoint defects in HeLa cells (Supplementary Fig. 1g). Similar synergy between Plk1 inhibition by BI 2536 and Bub1 depletion by siRNA-b or siRNA-c was observed in U2OS cells (Supplementary Fig. 1g). Therefore, our results reveal a critical role of Plk1 in the spindle checkpoint. Bub1 and Plk1 cooperate to maintain checkpoint-dependent mitotic arrest exerted by spindle poisons. The checkpoint becomes dependent on the kinase activity of Plk1 in cells with a compromised Bub1 function.

We tested whether the kinase activity of Bub1 was involved in this cooperation. Unlike Bub1 depletion, inhibition of the kinase activity of Bub1 with 2OH-BNPP1 did not synergize with Plk1 inhibition to produce checkpoint defects in HeLa cells^28^ (Supplementary Fig. 1h). 2OH-BNPP1 inhibited the kinase activity of Bub1 in these cells, as evidenced by the reduced phosphorylation of H2A, a known Bub1 substrate^40^ (Supplementary Fig. 1i). Furthermore, expression of the RNAi-resistant kinase-dead mutant of Bub1 rescued the checkpoint defects caused by Bub1 depletion and Plk1 inhibition (Fig. 1f,g). Thus, the cooperation between Bub1 and Plk1 involves the non-kinase domains of Bub1. Depletion of Bub1 by RNAi attenuates the function of Bub1 more severely than 2OH-BNPP1 does, as it reduces both the catalytic and scaffolding functions of Bub1.

### Bub1–Plk1 phosphorylates Cdc20 and inhibits APC/C^Cdc20^

Bub1 and Plk1 form a complex in mitosis, in a mechanism that requires the binding of the polo-box of Plk1 to a Cdk1-dependent phosphorylation site (T609) in Bub1 (ref. 32). Bub1 binds to Cdc20 through the Phe and KEN boxes^14^,^15^,^28^, and is required for the kinetochore localization of Cdc20 (ref. 14 and Supplementary Fig. 2a,b). Contrary to previous reports^14^,^41^, BubR1 depletion did not reduce Cdc20 kinetochore localization. Both Bub1 and BubR1 may contribute to Cdc20 kinetochore targeting and their relative contributions may vary in different cell lines.

**Figure 2:**
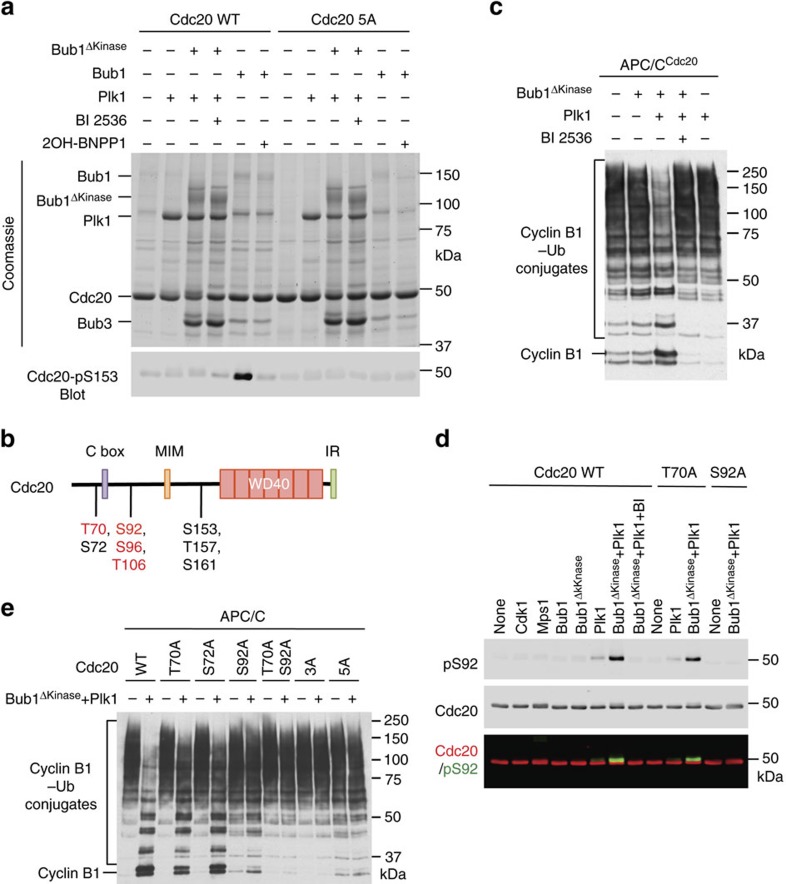
Bub1 promotes Plk1-mediated Cdc20 phosphorylation and APC/C inhibition. (**a**) Coomassie-stained gel (top) and Cdc20-pS153 blot (bottom) of kinase reactions containing the indicated recombinant proteins and inhibitors. Cdc20 5A, S72A/S92A/S153A/T157A/S161A. (**b**) Domains and motifs of human Cdc20 with the important phosphorylation sites indicated. Sites in Bub1–Plk1-treated Cdc20 identified by mass spectrometry in this study are labelled in red. IR, isoleucine–arginine tail; MIM, Mad2-interacting motif. (**c**) Anti-Myc blot of the *in-vitro* ubiquitination reactions of APC/C^Cdc20^ using cyclin B1_1–97_–Myc as the substrate. Cdc20 was first incubated with the kinase buffer in the presence or absence of indicated proteins and BI 2536 before being added to APC/C isolated from *Xenopus* egg extract. (**d**) Quantitative immunoblots of the kinase reactions containing the indicated recombinant kinases and Cdc20 proteins as substrates. BI 2536 (BI) was added to one of these reactions. The Cdc20-pS92 and total Cdc20 signals on the same membrane were detected in the 800- and 700-nm channels, respectively. The two channels were pseudo-coloured (Cdc20-pS92, green and Cdc20, red) and overlaid in the bottom panel. (**e**) Anti-Myc blot of the *in-vitro* ubiquitination reactions of APC/C^Cdc20^ using cyclin B1_1–97_–Myc as the substrate. The indicated Cdc20 proteins were first incubated with the kinase buffer in the presence and absence of Bub1^ΔKinase^ and Plk1, and then added to APC/C isolated from *Xenopus* egg extracts. Cdc20 3A, T70A/S72A/S92A.

We tested whether, through binding to both Plk1 and Cdc20 with distinct motifs, Bub1 might scaffold Plk1-dependent Cdc20 phosphorylation. As Bub1 could directly phosphorylate Cdc20 and because the kinase activity of Bub1 was not required for its functional cooperation with Plk1, we used the Bub1 truncation mutant lacking the kinase domain (Bub1^ΔKinase^) bound to Bub3 (to stabilize Bub1) in our assays. Bub1^Δkinase^–Bub3 was co-expressed with cyclin B1–Cdk1 to introduce phosphorylation at T609, which was required for Plk1 binding. Plk1 alone did not phosphorylate Cdc20 efficiently, as only a small fraction of Cdc20 underwent gel mobility shift (Fig. 2a). Addition of Bub1^ΔKinase^–Bub3 to this reaction caused ∼50% of Cdc20 to undergo gel mobility shift and this shift was abolished by BI 2536, suggesting that Bub1^ΔKinase^ stimulated Cdc20 phosphorylation by Plk1. Consistent with previous studies^27^,^28^,^42^, full-length Bub1 phosphorylated Cdc20 at S153. This phosphorylation was inhibited by 2OH-BNPP1 and did not retard the gel mobility of Cdc20. Even in the presence of Bub1^ΔKinase^, Plk1 did not phosphorylate Cdc20 S153, indicating that Plk1 and Bub1 phosphorylated different Cdc20 residues.

Several residues in the amino-terminal region of Cdc20 undergo phosphorylation in mitotic HeLa cells, including S72, S92, S153, T157 and S161 (Fig. 2b)^27^. The Cdc20 5A mutant with all five phospho-residues mutated to alanines had greatly reduced gel mobility shift caused by Bub1^ΔKinase^–Plk1 (Fig. 2a), suggesting that one or more of these mitotic phosphorylation events of Cdc20 might be mediated by Plk1.

Bub1-dependent phosphorylation of Cdc20 inhibits APC/C^27^. We tested whether phosphorylation of Cdc20 by Plk1 (as a component of Bub1–Plk1) also inhibited APC/C^Cdc20^. Whereas Bub1^ΔKinase^ or Plk1 alone did not appreciably inhibit APC/C^Cdc20^ (Fig. 2c), addition of both reduced the activity of APC/C^Cdc20^. BI 2536 reversed this inhibition. Thus, Bub1 stimulates Cdc20 phosphorylation by Plk1, which can inhibit APC/C^Cdc20^ *in vitro*.

We analysed Cdc20 phosphorylated by Bub1^ΔKinase^–Plk1 with mass spectrometry (Supplementary Fig. 2c,d) and identified several phosphorylation sites in Cdc20, including T70, S92, S96 and T106, with T70 and S92 being most prominent. We made phospho-specific antibodies against Cdc20-pT70 and Cdc20-pS92, and expressed and purified Cdc20 T70A and S92A proteins from Sf9 insect cells. Bub1^ΔKinase^ stimulated Plk1-dependent phosphorylation of Cdc20 at S92 (Fig. 2d). As controls, Bub1 and two other mitotic kinases, Cdk1 and Mps1, did not phosphorylate S92. The pS92 band co-migrated with the slow-migrating Cdc20 species (Fig. 2d) and S92A diminished the gel mobility shift of Cdc20. Phosphorylation of T70 was already detected in Cdc20 purified from Sf9 cells (Supplementary Fig. 2e). This residue is located in a Cdk1 consensus motif, suggesting that it might be phosphorylated by Cdk1 in insect cells. Indeed, among the three mitotic kinases tested, only Cdk1 could phosphorylate T70 on recombinant Cdc20 that had been dephosphorylated by λ phosphatase (Supplementary Fig. 2f). T70 is thus not a Plk1 site. The T70A mutation did not affect S92 phosphorylation by Bub1^ΔKinase^–Plk1 (Fig. 2d), indicating that phosphorylation of T70 is not a prerequisite for S92 phosphorylation. Our results establish Cdc20 S92 as a major Plk1 phosphorylation site *in vitro*.

We tested whether the phospho-deficient Cdc20 mutants were refractory to Bub1^ΔKinase^–Plk1 inhibition. We expressed and purified additional Cdc20 mutants, including S72A, T70A/S92A, T70A/S72A/S92A (3A) and 5A. Compared with Cdc20 wild type (WT), T70A and S72A, Cdc20 S92A was much less efficiently inhibited by Bub1^ΔKinase^–Plk1 (Fig. 2e). On the other hand, Cdc20 S92A was still slightly inhibited by Bub1^ΔKinase^–Plk1, whereas Cdc20 5A was completely resistant to Bub1^ΔKinase^–Plk1 inhibition (Fig. 2e), indicating Bub1^ΔKinase^–Plk1 might phosphorylate additional sites aside from S92. We had previously shown that Cdc20 5A was resistant to Bub1 inhibition^27^. Therefore, we have identified the major phosphorylation sites of Bub1–Plk1 on Cdc20 that are functionally important for APC/C^Cdc20^ inhibition *in vitro*.

### Bub1/BubR1–Plk1 mediate Cdc20 phosphorylation

**Figure 3:**
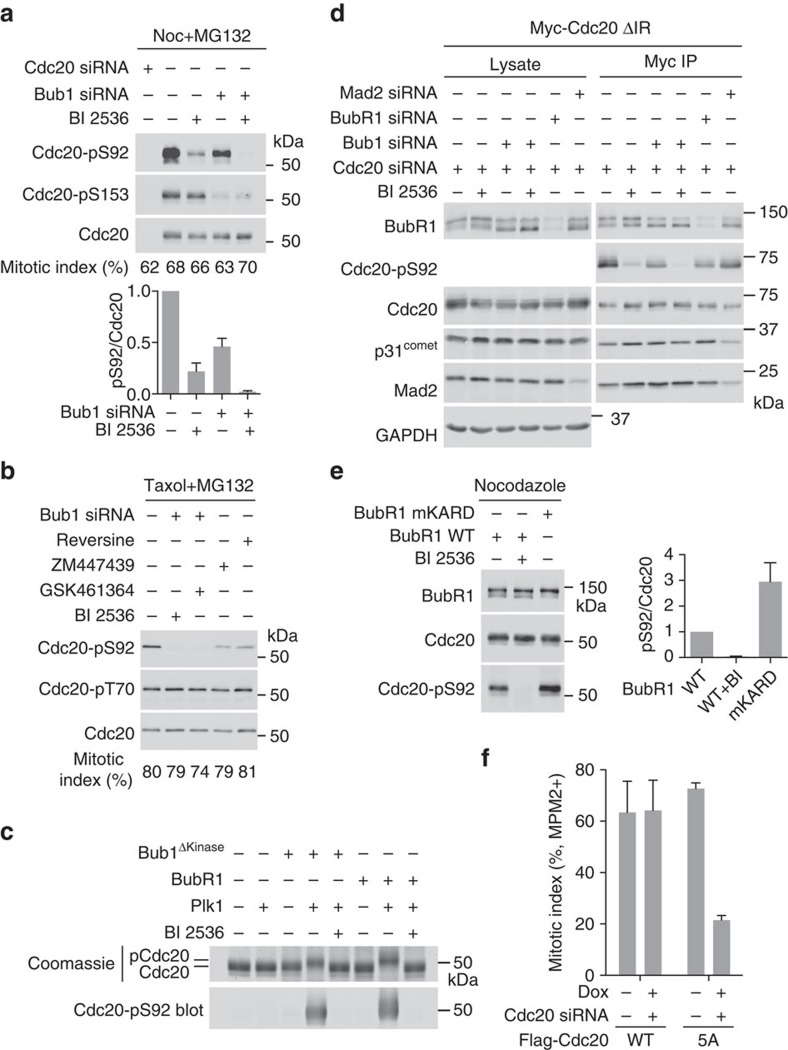
Cdc20 phosphorylation by Bub1–Plk1 and BubR1–Plk1 is regulated by and required for the spindle checkpoint. (**a**) HeLa Tet-On cells were treated with the indicated siRNAs, arrested in mitosis using 500 nM nocodazole (Noc) and 10 μM MG132, and then treated with or without BI 2536. The endogenous Cdc20 was immunoprecipitated from these cells and blotted with the indicated antibodies. The mitotic index of each sample is indicated below each lane. The bottom graph shows the quantification of the Cdc20-pS92 signal normalized to the total Cdc20 signal (mean±range; *n*=2 independent experiments). (**b**) HeLa Tet-On cells were treated with or without Bub1 siRNA and arrested in mitosis by 200 nM taxol and 10 μM MG132, and then incubated with the indicated kinase inhibitors. The endogenous Cdc20 was immunoprecipitated from these cells and blotted with the indicated antibodies. The mitotic index of each sample is indicated below each lane. (**c**) Coomassie-stained gel (top) and Cdc20-pS92 blot (bottom) of the kinase reactions of recombinant Cdc20 and other indicated proteins. (**d**) HeLa Tet-On cells stably expressing Myc-Cdc20 ΔIR were treated with the indicated siRNAs and 500 nM nocodazole, and then treated with or without BI 2536. Cell lysates and anti-Myc immunoprecipitates (IP) were blotted with indicated antibodies. The Cdc20-pS92 antibody could not detect S92 phosphorylation in cell lysates. (**e**) HeLa Tet-On cells were transfected with BubR1 siRNA and plasmids encoding siRNA-resistant Myc-BubR1 WT or the KARD motif mutant (mKARD), arrested in mitosis with 500 nM nocodazole (Noc) and treated with or without BI 2536. Endogenous Cdc20 was immunoprecipitated from these cells and the IP was blotted with indicated antibodies. Right graph shows the quantification of the Cdc20-pS92 signal normalized to that of total Cdc20 (mean±range; *n*=2 independent experiments). (**f**) Quantification of the mitotic index of HeLa Tet-On cells stably expressing Flag-Cdc20 WT or 5A treated with or without doxycycline (Dox) or Cdc20 siRNA, and then incubated with 500 nM nocodazole. Mean±s.d.; *n*=3 independent experiments.

We tested whether Bub1–Plk1 phosphorylated Cdc20 in human cells. The proteasome inhibitor MG132 was added to prevent mitotic exit of cells with an inactive spindle checkpoint. Endogenous Cdc20 was indeed phosphorylated at S92 and S153 in mitosis (Fig. 3a). Bub1 depletion expectedly reduced S153 phosphorylation. Either BI 2536 or Bub1 depletion reduced S92 phosphorylation and their combination abolished this phosphorylation (Fig. 3a). Inhibition of Plk1 by GSK461364 together with Bub1 depletion also abolished S92 phosphorylation (Fig. 3b). Thus, Bub1–Plk1 phosphorylates Cdc20 during mitosis in human cells.

BubR1 can also bind to Plk1 through a phosphorylated STP motif and Cdc20 through Phe and D boxes in the middle region^15^,^43^. Indeed, recombinant purified BubR1 stimulated Plk1-dependent phosphorylation of Cdc20 at S92 (Fig. 3c), indicating that BubR1 could also scaffold Plk1-mediated phosphorylation of Cdc20. As BubR1 was critical for the spindle checkpoint, we could not effectively synchronize cells depleted of BubR1 in mitosis. We thus constructed a HeLa cell line stably expressing a Cdc20 mutant with its carboxy-terminal IR motif deleted (Cdc20 ΔIR). Cdc20 ΔIR could not bind to or activate APC/C but retained all other functional motifs^44^,^45^,^46^. Depletion of endogenous Cdc20 with siRNA arrested Cdc20 ΔIR-expressing cells in mitosis in a checkpoint-independent manner, allowing us to synchronize cells depleted of BubR1 or Mad2 in mitosis. Similar to Bub1 depletion, depletion of BubR1 reduced S92 phosphorylation on Cdc20 ΔIR (Fig. 3d). As a control, depletion of Mad2 did not affect this phosphorylation. Thus, BubR1–Plk1 can mediate Plk1-dependent phosphorylation of Cdc20 in human cells. Furthermore, Cdc20 ΔIR still bound to Mad2 and BubR1 in cells depleted of Bub1 and treated with BI 2536 (Fig. 3d). As these cells had much reduced levels of Cdc20 S92 phosphorylation, Cdc20 phosphorylation might not be required for MCC formation.

BubR1 interacts with both Cdc20 and PP2A through neighbouring motifs^15^,^34^. The Cdc20-pS92 level of cells overexpressing the BubR1 mutant with its KARD motif mutated was about threefold that of cells expressing WT BubR1 (Fig. 3e). Thus, BubR1–Plk1 may also contribute to Cdc20 phosphorylation in human cells, but this mechanism may be limited by BubR1-bound PP2A.

The kinase activities of Mps1 and Aurora B are required for spindle checkpoint signalling in taxol-treated human cells. The Mps1 inhibitor reversine and the Aurora B inhibitor ZM447439 reduced S92 phosphorylation in mitotic cells (Fig. 3b). As a control, Cdc20 T70 phosphorylation was not affected. Furthermore, when Aurora B was inhibited to different levels with different doses of ZM447439, the level of S92 phosphorylation correlated with the Aurora B activity and possibly checkpoint strength (Supplementary Fig. 3a). Thus, S92 phosphorylation is sensitive to checkpoint status. On the other hand, this phosphorylation is not completely eliminated when the checkpoint is off, indicating that it is not strictly dependent on the checkpoint. A pool of Cdc20 can be phosphorylated by Plk1 in mitosis, regardless of checkpoint status.

Expression of the phospho-deficient Cdc20 5A, but not Cdc20 WT, in cells depleted of endogenous Cdc20 caused these cells to escape from nocodazole-mediated mitotic arrest (Fig. 3f). Mutating either S92 or S153 to alanine alone did not result in checkpoint defects, even with Bub1 siRNA or BI 2536 treatment (Supplementary Fig. 3b), indicating that these two sites were not the only functional Bub1–Plk1 sites in Cdc20. We constructed a 4A mutant (S84A/S92A/S96A/S153A), because S84 and S96 were adjacent to S92 and were phosphorylated *in vitro* (Supplementary Fig. 2c,d). The 4A mutant still did not produce spindle checkpoint deficiency. We conclude that Bub1–Plk1 phosphorylates multiple sites in Cdc20. Aside from S92 and S153, one or more residues among S72, T157 and S161 might also be functionally important sites for the kinase complex. Taken together, these results indicate that Bub1–Plk1-dependent phosphorylation of Cdc20 is regulated by upstream checkpoint signalling and is required for checkpoint-dependent mitotic arrest in human cells. Compared with cells treated with other siRNAs, cells treated with Bub1 siRNA-d had a slightly lower Plk1 protein level in cell lysates (Supplementary Fig. 3c), which might help to explain the checkpoint deficiency produced by Bub1 siRNA-d.

### Bub1 scaffolds Cdc20 phosphorylation

**Figure 4:**
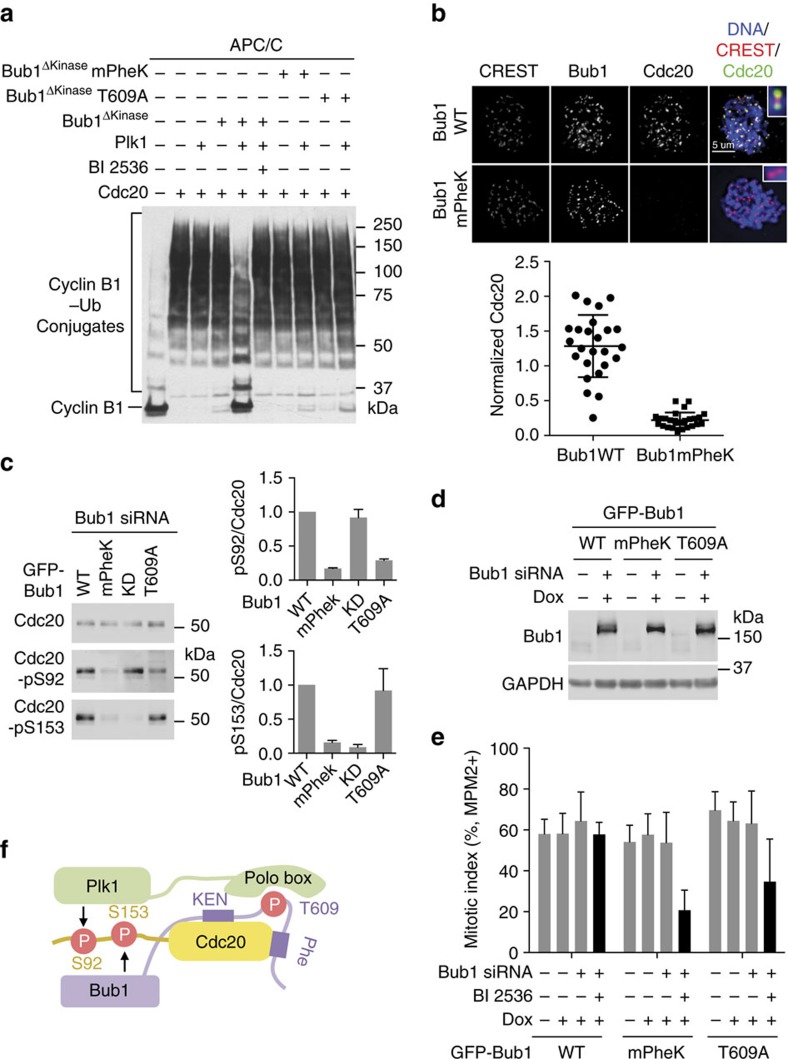
Bub1 acts as a scaffold to promote Plk1-mediated Cdc20 phosphorylation and APC/C^Cdc20^ inhibition. (**a**) Anti-Myc blot of the *in-vitro* ubiquitination reactions of APC/C^Cdc20^ using cyclin B1_1–97_–Myc as the substrate. Cdc20 was first incubated in the kinase buffer in the presence or absence of indicated proteins and BI 2536 before being added to APC/C isolated from *Xenopus* egg extract. Bub1^ΔKinase^ mPheK, the Bub1^ΔKinase^ mutant with the Phe and KEN boxes mutated, which contains the K535A, E536A, N537A, K625A, E626A and N627A mutations. (**b**) HeLa Tet-On cells stably expressing GFP-Bub1 WT or mPheK were treated with Bub1 siRNA and arrested in mitosis with 250 nM nocodazole. Cells were stained with 4,6-diamidino-2-phenylindole (DAPI) and the indicated antibodies. Colours of the overlaid channels match those of the label. Selected regions were magnified and shown in insets. Scale bar, 5 μm. The bottom graph shows the quantification of the Cdc20 kinetochore staining intensity normalized to that of CREST (mean±s.d.; each dot represents one cell). (**c**) HeLa Tet-On cells stably expressing GFP-Bub1 WT or mutants were treated with Bub1 siRNA and 500 nM nocodazole. The endogenous Cdc20 was immunoprecipitated from these cells and blotted with the indicated antibodies. Graphs on the right show the quantification of Cdc20-pS92 (top) or Cdc20-pS153 (bottom) signals normalized to that of total Cdc20 (mean±range; *n*=2 independent experiments). (**d**) HeLa Tet-On cells stably expressing GFP-Bub1 WT or mutants were treated with or without Bub1 siRNA and doxycycline (Dox), and arrested in mitosis with 500 nM nocodazole. Cell lysates were blotted with the indicated antibodies. (**e**) Quantification of the mitotic index of HeLa Tet-On cells stably expressing GFP-Bub1 WT, mPheK or T609A treated with 200 nM taxol and the indicated siRNA and compounds (mean±s.d.; *n*=3 independent experiments). Data of GFP-Bub1 WT in this figure were the same as those in Fig. 1g. (**f**) Model explaining how the non-kinase domains of Bub1 act as a scaffold to promote Cdc20 phosphorylation by both the kinase domains of Bub1 and Plk1.

We tested which Bub1 motifs were critical for Bub1^ΔKinase^–Plk1-dependent inhibition of APC/C^Cdc20^. The Phe and KEN boxes of Bub1 bind cooperatively to Cdc20 (refs 14, 15, 28). A Bub1 mutant with both motifs mutated (mPheK) is deficient in Cdc20 binding^28^. As Plk1 binding to Bub1 requires phosphorylation of T609 in Bub1, Bub1 T609A is deficient in Plk1 binding^32^. Both Bub1 mPheK and T609A failed to support Plk1-dependent inhibition of APC/C^Cdc20^ (Fig. 4a). Therefore, binding of both Cdc20 and Plk1 by Bub1 is required for APC/C^Cdc20^ inhibition.

Ectopic expression of Bub1 WT, but not mPheK, in Bub1 RNAi cells restored Cdc20 to kinetochores (Fig. 4b), indicating that the Bub1–Cdc20 interaction is required for the kinetochore targeting of Cdc20. Consistent with previous studies^28^, Bub1 mPheK failed to support Bub1-dependent phosphorylation of Cdc20 S153 (Fig. 4c). Bub1 T609A was functional in supporting S153 phosphorylation, confirming that Plk1 does not phosphorylate this site. Both Bub1 mPheK and T609A were deficient in supporting S92 phosphorylation, with mPheK being more defective (Fig. 4c). The Bub1 kinase-dead mutant fully supported S92 phosphorylation. Finally, we created stable HeLa cell lines that inducibly expressed GFP-Bub1 WT, mPheK or T609A (Fig. 4d). Consistent with their inability to support Cdc20 phosphorylation, both Bub1 mPheK and T609A were defective in restoring nocodazole-induced mitotic arrest in these cells depleted of endogenous Bub1 and treated with BI 2536 (Fig. 4e). Bub1 T609A was less defective than mPheK, presumably because Bub1 T609A still supported Bub1-dependent phosphorylation of Cdc20, including S153.

Taken together, our results are consistent with the following mechanism for Bub1–Plk1-dependent Cdc20 phosphorylation (Fig. 4f). Bub1 binds to Cdc20 and Plk1 through distinct functional motifs, forming a transient Cdc20–Bub1–Plk1 complex. The kinase domains of both Plk1 and Bub1 then phosphorylate different sets of residues in the N-terminal region of Cdc20, including S92 and S153. As Bub1 is required for the kinetochore localization of both Cdc20 and Plk1, these phosphorylation events may occur at kinetochores. Cdc20 phosphorylation by Bub1–Plk1 inhibits APC/C^Cdc20^ and contributes to spindle checkpoint signalling.

### Phospho-mimicking Cdc20 mutations suffice to inhibit APC/C

**Figure 5:**
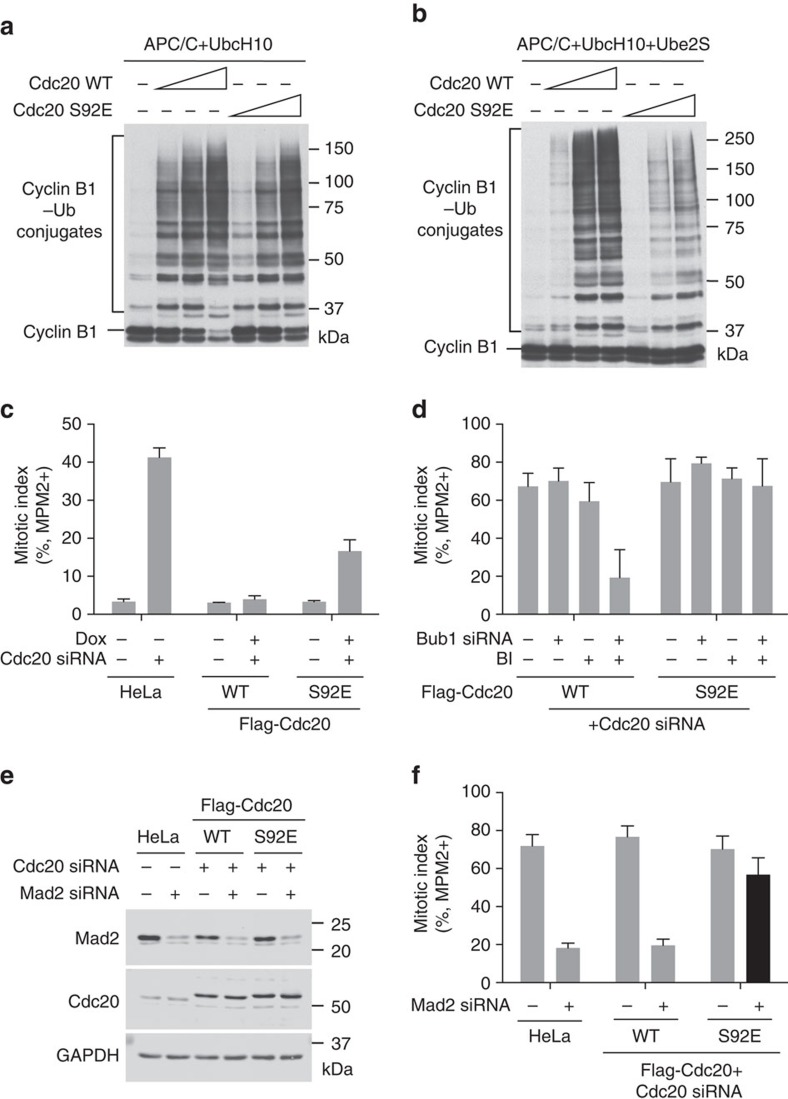
The Cdc20 phospho-mimicking mutant S92E is defective in APC/C activation. (**a**,**b**) Anti-Myc blot of the *in-vitro* ubiquitination reactions of APC/C^Cdc20^ using cyclin B1_1–97_–Myc as the substrate and UbcH10 (**a**) or both UbcH10 and Ube2S (**b**) as ubiquitin-conjugating enzymes. Recombinant Cdc20 WT or S92E at different concentrations (16.6, 66.4 and 332 nM) were incubated with APC/C isolated from *Xenopus* egg extract. (**c**) Quantification of the mitotic index of HeLa Tet-On parental cells and cells stably expressing Flag-Cdc20 WT or S92E that were treated with or without Cdc20 siRNA or Dox (mean±s.d.; *n*=3 independent experiments). (**d**) Quantification of the mitotic index of HeLa Tet-On cells stably expressing Flag-Cdc20 WT or S92E that were treated with Cdc20 siRNA, Dox and 200 nM taxol in the presence or absence of Bub1 siRNA or BI 2536 (BI) (mean±s.d.; *n*=3 independent experiments). (**e**) HeLa Tet-On parental cells and cells stably expressing Flag-Cdc20 WT or S92E were treated with the indicated siRNAs and 500 nM nocodazole. Cell lysates were blotted with the indicated antibodies. (**f**) Quantification of the mitotic index of cells in **e** (mean±s.d.; *n*=3 independent experiments).

We made the phospho-mimicking Cdc20 S92E mutant and tested its ability to activate APC/C *in vitro*. UbcH10 initiates ubiquitin chain assembly on APC/C substrates and Ube2S then elongates these ubiquitin chains^47^. Cdc20 S92E was less effective than WT to activate APC/C-dependent ubiquitination of cyclin B1 in the presence of UbcH10 alone (Fig. 5a). The activity difference between Cdc20 WT and S92E was greater when both UbcH10 and Ube2S were used (Fig. 5b). Thus, Cdc20 S92E is deficient in activating APC/C and has a strong effect on Ube2S-dependent elongation of ubiquitin chains. This result indicates that Cdc20 S92E can functionally mimic the effects of Plk1-dependent phosphorylation, at least to some degree. The S153E mutant of Cdc20, which mimicked a key Bub1 phosphorylation event, was also less active (Supplementary Fig. 4a). Combining the two phospho-mimicking mutations further reduced Cdc20 activity, supporting our conclusion that Bub1–Plk1 collaborate to phosphorylate Cdc20 and prevent it from activating APC/C.

We made stable HeLa cell lines that inducibly expressed RNAi-resistant Flag-Cdc20 WT or S92E. Depletion of Cdc20 from parental HeLa cells by RNAi produced mitotic arrest in the absence of spindle poisons^48^ (Fig. 5c). Expression of Cdc20 WT rescued the mitotic arrest phenotype caused by Cdc20 depletion. Consistent with its weaker APC/C-stimulatory activity *in vitro*, Cdc20 S92E only partially rescued the mitotic arrest phenotype (Fig. 5c). Moreover, about 25% of HeLa cells treated with nocodazole underwent mitotic adaptation or slippage after a prolonged mitotic arrest^49^, as indicated by the population of cells that had 4N DNA content but were negative for MPM2 staining (Supplementary Fig. 4b). Expression of Cdc20 S92E, but not WT, in cells depleted of endogenous Cdc20 greatly reduced mitotic adaptation in the presence of nocodazole (Supplementary Fig. 4b,c). Thus, Cdc20 S92E is defective in activating APC/C^Cdc20^ in human cells. Finally, expression of S92E restored nocodazole-induced mitotic arrest in cells depleted of Bub1 and Cdc20, and treated with BI 2536 (Fig. 5d). This result suggests that phosphorylation of Cdc20 S92 is a major function of Plk1 in the spindle checkpoint.

To explore the relationship between MCC and Plk1-dependent Cdc20 phosphorylation in APC/C^Cdc20^ inhibition, we examined the phenotypes of Cdc20 S92E-expressing cells depleted of Mad2 or BubR1. Strikingly, the phospho-mimicking Cdc20 S92E restored nocodazole-induced mitotic arrest of cells partially depleted of Mad2 or BubR1 (Fig. 5e,f and Supplementary Fig. 4d). Although we cannot exclude the possibility that this phospho-mimicking mutation might have effects other than mimicking a phosphorylation event, this result suggests that phosphorylation of Cdc20 might inhibit APC/C^Cdc20^ in a mechanism parallel to MCC-dependent inhibition of APC/C^Cdc20^. Alternatively, Cdc20 S92 phosphorylation might enhance the activity of the residual MCC in Mad2- or BubR1-depleted cells.

### Cdc20 phosphorylation is dispensable for MCC formation

**Figure 6:**
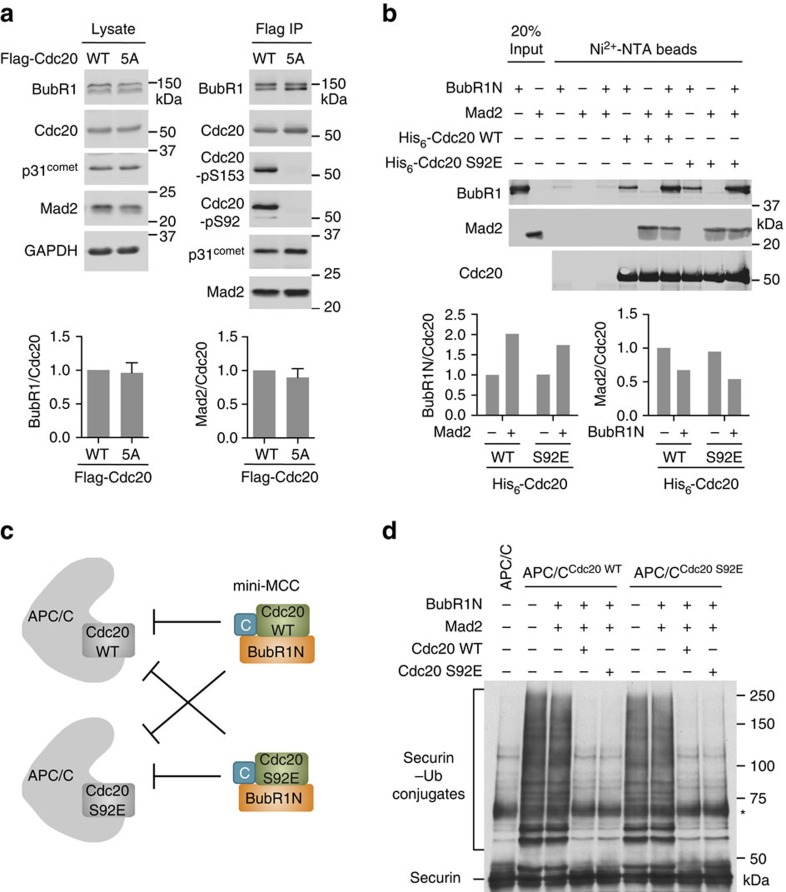
Cdc20 phosphorylation by Bub1–Plk1 is dispensable for MCC formation. (**a**) HeLa Tet-On cells stably expressing Flag-Cdc20 WT or 5A were arrested in mitosis by 5 μM nocodazole. The cell lysates and the anti-Flag immunoprecipitates (IP) of these cells were blotted with the indicated antibodies. The graphs at the bottom show the quantification of the relative BubR1 and Mad2 signals normalized to that of total Cdc20 in the IP (mean±range; *n*=2 independent experiments). (**b**) Blots of the input and beads-bound proteins of the binding reactions among the indicated proteins. The graphs at the bottom show the quantification of the relative BubR1 and Mad2 signals normalized to Cdc20. (**c**) Schematic drawing of the experimental design in **d** and Supplementary Fig. 4b. APC/C is pre-activated with Cdc20 WT or S92E and then incubated with mini-MCC comprising BubR1N, Mad2 in the closed conformation and Cdc20 (WT or S92E). (**d**) Anti-Myc blot of the *in-vitro* ubiquitination reactions of the indicated APC/C^Cdc20^ incubated with the indicated proteins and using securin-Myc as the substrate. The asterisk indicates a nonspecific cross-reacting band.

We immunoprecipitated Cdc20 WT and 5A from HeLa cells stably expressing them, and examined their association with BubR1 and Mad2. Cdc20 5A bound to BubR1 and Mad2 as efficiently as Cdc20 WT did (Fig. 6a). This result suggests that Cdc20 phosphorylation is not required for MCC assembly in human cells. We next reconstituted a mini-MCC with recombinant human Mad2, Cdc20 and an N-terminal fragment of BubR1 (BubR1N; residues 1–370). In the absence of Mad2, BubR1N bound weakly to Cdc20 (Fig. 6b). Addition of Mad2 greatly stimulated BubR1N binding to Cdc20, indicative of the formation of the BubR1N–Cdc20–Mad2 complex (mini-MCC). The phospho-mimicking Cdc20 S92E did not exhibit increased binding to Mad2 or BubR1N. Thus, Cdc20 phosphorylation at S92 does not stimulate MCC formation *in vitro*.

The Mad2 inhibitor p31^comet^ promotes MCC disassembly through multiple mechanisms, including stimulating Cdc20 autoubiquitination^45^,^50^,^51^,^52^,^53^,^54^,^55^,^56^,^57^. The Cdc20 5A mutation did not appreciably alter p31^comet^ binding to Cdc20 (Fig. 6a). Cdc20 S92E still underwent autoubiquitination *in vitro* (Supplementary Fig. 5a). Therefore, we have no evidence to indicate that Cdc20 phosphorylation can regulate MCC disassembly.

Although Cdc20 phosphorylation appear to be dispensable for MCC assembly, it remains possible that it might enhance the APC/C-inhibitory activity of MCC. MCC not only sequesters one Cdc20 molecule but also inhibits another Cdc20 molecule that is already bound to APC/C^26^. We examined the effect of S92E on MCC-mediated inhibition of APC/C^Cdc20^. First, we activated APC/C with Cdc20 WT and then tested its inhibition by mini-MCC containing either Cdc20 WT or S92E (Fig. 6c), with either securin (Fig. 6d) or cyclin B1 (Supplementary Fig. 5b) as the substrate. Mad2 and BubR1N did not inhibit the already activated APC/C^Cdc20^. Addition of Cdc20 along with Mad2 and BubR1N led to the formation of mini-MCC, which inhibited the already activated APC/C^Cdc20^ (Fig. 6d and Supplementary Fig. 5b). Importantly, mini-MCC containing Cdc20 S92E did not inhibit APC/C bound to Cdc20 WT more efficiently than mini-MCC containing Cdc20 WT did.

Second, we activated APC/C with Cdc20 S92E and then tested its inhibition by mini-MCC containing Cdc20 WT or S92E (Fig. 6c,d and Supplementary Fig. 5b). As Cdc20 S92E was less active in stimulating APC/C, we used higher doses of this mutant to normalize the activities of APC/C bound to Cdc20 WT and S92E. Again, Cdc20 S92E did not enhance MCC-mediated inhibition of APC/C^Cdc20^, even when both copies of Cdc20 contained the phospho-mimicking mutation (Fig. 6d and Supplementary Fig. 4b). Thus, Cdc20 phosphorylation does not appear to stimulate the APC/C-inhibitory activity of MCC. Taken together, our results suggest that Cdc20 phosphorylation by Bub1–Plk1 directly inhibits APC/C^Cdc20^ and acts in a mechanism that is parallel, but not redundant, to MCC formation.

## Discussion

A few unattached kinetochores within a human cell are capable of activating the spindle checkpoint and delaying the onset of anaphase^58^,^59^,^60^. The current model of spindle checkpoint signalling posits that these unattached kinetochores produce diffusible wait-anaphase entities to inhibit APC/C^Cdc20^ throughout the cell. MCC is a critical APC/C inhibitor in the spindle checkpoint, and is likely to be a diffusible wait-anaphase entity. On the other hand, MCC involves the physical binding of BubR1 and Mad2 to Cdc20 and is thus a stoichiometric inhibitor of APC/C^Cdc20^. It is unclear whether a few unattached kinetochores are sufficient to produce enough MCC to inhibit all cellular APC/C.

In this study, we establish Cdc20 phosphorylation by the heterodimeric Bub1–Plk1 kinase complex as another critical APC/C-inhibitory mechanism in the spindle checkpoint. The kinase domains of both Bub1 and Plk1 in this complex can directly phosphorylate distinct residues in the N-terminal region of Cdc20. Bub1 interacts with Cdc20 through the Phe and KEN motifs, and with Plk1 through a phosphorylated STP motif. The Bub1–Cdc20 interaction promotes phosphorylation of Cdc20 by both Bub1 and Plk1, whereas Plk1-mediated phosphorylation of Cdc20 additionally requires the Bub1–Plk1 interaction. Thus, Bub1 not only phosphorylates Cdc20 as a kinase, but also facilitates Cdc20 phosphorylation by Plk1 as a scaffold. Our biochemical and functional analyses further establish Cdc20 phosphorylation by Bub1–Plk1 as a functionally relevant, catalytic mechanism for checkpoint-dependent inhibition of APC/C.

The biochemical mechanism by which Cdc20 phosphorylation inhibits APC/C^Cdc20^ is unknown at present. Several phosphorylation sites are located in close proximity to the C box and the Mad2-interacting motif, which mediate the productive binding of Cdc20 to APC/C^61^,^62^. It is possible that phosphorylation of Cdc20 might alter the mode of Cdc20 binding to APC/C or anchor Cdc20 at an APC/C site that is not conducive to catalysis. Furthermore, as Cdc20 S92E was particularly defective in supporting Ube2S-mediated ubiquitination, this phosphorylation might also regulate the interaction between Cdc20 and Ube2S.

We had previously shown that Cdc20 phosphorylation by Bub1 inhibited APC/C^Cdc20^ and proposed that Cdc20 phosphorylation by Bub1 provided a catalytic mechanism for checkpoint-dependent inhibition of APC/C^27^. Subsequent studies, however, showed that the kinase activity of Bub1 was not strictly required for the spindle checkpoint in human cells^28^,^29^. Mouse cells with the kinase activity of Bub1 genetically inactivated had mild spindle checkpoint defects that were attributed to the function of Bub1 in phosphorylating histone H2A and installing Aurora B at centromeres^30^. These results casted doubts on the relevance of Cdc20 phosphorylation by Bub1 in checkpoint signalling and suggested that the non-kinase domains of Bub1 might play important roles in the spindle checkpoint. Indeed, the non-kinase region of Bub1 interacts with BubR1–Bub3, Mad1–Mad2 and Cdc20 through distinct conserved motifs in human and yeast cells^14^,^28^,^63^,^64^,^65^. An emerging picture is that Bub1 acts as a scaffold to promote the kinetochore targeting of other checkpoint proteins. On the other hand, a very small amount of Bub1 in human cells suffices to promote checkpoint signalling, a notion seemingly at odds with a simple scaffolding role of Bub1.

Our study now clarifies the roles of the kinase and non-kinase domains of Bub1, and resolves these long-standing conundrums in the field. Our results show that the non-kinase domains of Bub1 recruit Plk1 and enable Plk1-dependent phosphorylation of Cdc20 and inhibition of APC/C^Cdc20^. Thus, the non-kinase domains of Bub1 have an unconventional ‘catalytic' role indirectly through the associated kinase Plk1, reconciling why the kinase activity of Bub1 is not strictly required for the checkpoint and yet a small amount Bub1 is sufficient to maintain checkpoint signalling. As this heterodimeric Bub1–Plk1 kinase complex has two catalytic activities with partially redundant functions in APC/C^Cdc20^ inhibition, it is difficult to inactivate this catalytic engine of the checkpoint in human cells. Only the combination of chemical inhibition of Plk1 and depletion of Bub1 can substantially weaken both activities and produce strong checkpoint defects.

BubR1 also stimulates Cdc20 phosphorylation by Plk1 *in vitro* and may contribute to Cdc20 phosphorylation in human cells. On the other hand, Plk1-dependent phosphorylation of the KARD motif in BubR1 enables the binding of PP2A^34^. BubR1-bound PP2A might suppress Cdc20 phosphorylation by Plk1.

Several lines of evidence suggest that Cdc20 phosphorylation by Bub1–Plk1 acts in a pathway parallel (but not redundant) to MCC formation in APC/C inhibition. First, Cdc20 phosphorylation by Bub1–Plk1 directly inhibits APC/C^Cdc20^ *in vitro*, in the absence of Mad2 and BubR1. Second, the Cdc20 mutant deficient for Bub1–Plk1 phosphorylation is capable of forming MCC in human cells. Third, the phospho-mimicking Cdc20 S92E mutation lessens the requirement for Mad2 or BubR1 in the spindle checkpoint in human cells, without affecting MCC formation or activity *in vitro*. On the other hand, our results do not rule out the possibility that Cdc20 phosphorylation contributes to MCC formation or activity *in vivo*, where additional regulators of this process are present.

MCC is constantly assembled and disassembled even when checkpoint is on. It is possible that not all Cdc20 is bound and inhibited by MCC. Indeed, a population of Cdc20 was not associated with MCC in the presence of an active checkpoint^66^. We propose that although MCC may sequester and inhibit the bulk of Cdc20 when the checkpoint is on, Cdc20 phosphorylation is required to prevent a population of free Cdc20 from prematurely activating APC/C. When the checkpoint needs to be turned off, PP2A or other phosphatases can quickly dephosphorylate Cdc20 and allow APC/C activation, whereas MCC disassembly may be a relatively slow process.

The spindle checkpoint is deficient in cells partially depleted of Mad2 or BubR1. Yet, ectopic expression of Cdc20 S92E rescues the checkpoint defect of these cells. An interesting question is why Bub1–Plk1-dependent phosphorylation of the endogenous Cdc20 cannot sustain checkpoint signalling in cells partially depleted of Mad2 or BubR1. One possibility is that not all Cdc20 is phosphorylated at the S92 site. PP2A-dependent mechanisms might actively suppress the extent of Cdc20 phosphorylation, even when upstream checkpoint signalling is on. However, the phospho-mimicking S92E mutant (with the endogenous Cdc20 depleted) mimics the phosphorylation of most Cdc20 molecules, a scenario not likely to be attainable with the existence of opposing phosphatases.

**Figure 7:**
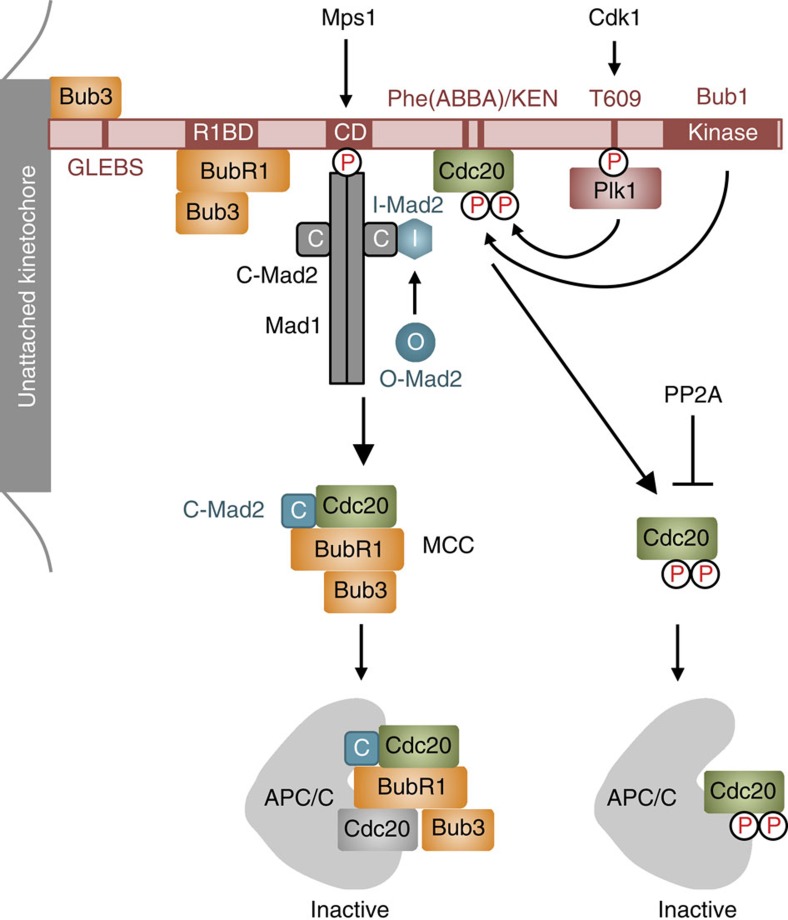
Model explaining the relationship between MCC and Cdc20 phosphorylation by Bub1–Plk1 in checkpoint-dependent inhibition of APC/C. In this model, Bub1 acts as a scaffold at kinetochores to recruit Plk1 and all components of MCC. It promotes MCC formation and Cdc20 phosphorylation by both Bub1 and Plk1, which represent two parallel, but not redundant, mechanisms to inhibit APC/C. Both mechanisms are required to maintain checkpoint signalling. Either mechanism is insufficient to sustain the checkpoint. CD, conserved domain; GLEBS, Gle2-binding sequence; R1BD, BubR1-binding domain.

We propose the following Bub1 scaffolding model for spindle checkpoint signalling (Fig. 7). During mitosis, the constitutive Bub1–Bub3 complex is phosphorylated by Cdk1 at T609, binds to Plk1, and is recruited to kinetochores. Bub1–Bub3 further recruits BubR1–Bub3, Mad1–Mad2 and Cdc20 to kinetochores. In one mechanism, through its physical interactions with all components of MCC, Bub1 promotes the formation of MCC, which can inhibit free APC/C or APC/C already bound to Cdc20. In a parallel but not redundant mechanism, Bub1 and Bub1-bound Plk1 can both phosphorylate Cdc20 and inhibit APC/C^Cdc20^ catalytically. Both MCC formation and Cdc20 phosphorylation are required for proper spindle checkpoint signalling. Inactivation of either mechanism produces checkpoint defects and can result in chromosome missegregation and aneuploidy.

## Methods

### Cell culture and transfection

HeLa Tet-On (Clontech) and U2OS (ATCC) cells were cultured in DMEM medium (Life Technologies) with 10% fetal bovine serum (Life Technologies) and 10 mM L-glutamine (Life Technologies). For cell cycle arrest in mitosis, cells were incubated in medium containing 2.5 mM thymidine (Sigma) for 16 h, released into fresh medium for 7 h and incubated in medium containing nocodazole (500 nM or 5 μM; Sigma) or taxol (200 nM; Sigma) for another 3–5 h. Inhibitors were added at 1–3 h before sample collection. Inhibitors used in this study were as follows: the Aurora kinase inhibitor ZM447439 (used at 2 μM; Selleck Chemicals), the Bub1 kinase inhibitor 2OH-BNPP1 (used at 4 μM), the Plk1 kinase inhibitors BI 2536 (used at 100 nM; Selleck Chemicals) and GSK461364 (used at 200 nM; Selleck Chemicals), the Mps1 kinase inhibitor reversine (used at 100 nM; Cayman Chemical) and the proteasome inhibitor MG132 (used at 10 μM; Boston Biochem). For the BI 2536 and GSK461364 titration experiment, various concentrations of the two inhibitors were added to log-phase cells. Cells were harvested after a 16-h incubation.

Transfection of siRNAs and plasmids was performed using Lipofectamine RNAiMAX (Life Technologies) and Effectene (QIAGEN), respectively, following the manufacturers' protocols. A final concentration of 5 nM per siRNA was used, unless otherwise stated. The Cdc20 siRNA was the Silencer Select Pre-designed siRNA from Ambion (ID s2758) with the following sequence: 5′-CGAAAUGACUAUUACCUGA-3′. All other siRNAs were synthesized at Thermo Scientific. The sequences of these siRNAs were as follows: Luciferase (Luc) siRNA, 5′-UCAUUCCGGAUACUGCGAU-3′; Mps1 siRNA, 5′-UGAACAAAGUGAGAGACAU-3′; Bub1 siRNA-b, 5′-CCAUGGGAUUGGAACCCUG-3′; Bub1 siRNA-c, 5′-CCCAUUUGCCAGCUCAAGC-3′; Bub1 siRNA-d, 5′-GAGUGAUCACGAUUUCUAA-3′ (ref. 29); Bub1 siRNA-f, 5′-GGCAAAAGCUGAAGAAAGU-3′; Bub1 siRNA-h, 5′-GAAACGGAUUUUUGGAACA-3′; Ndc80 siRNA, 5′-GAGUAGAACUAGAAUGUGA-3′; BubR1 siRNA, 5′-CAAGAUGGCUGUAUUGUUU-3′; and Mad2 siRNA, 5′-UACGGACUCACCUUGCUUG-3′.

For the generation of stable cell lines, HeLa Tet-On cells were transfected with pTRE2hygro vectors encoding GFP-Bub1 WT or mutants (resistant to Bub1 siRNA-b, -c and -d), Flag-Cdc20 WT or mutants, or Myc-Cdc20 ΔIR (resistant to Cdc20 siRNA from Ambion) and selected with 400 μg ml^−1^ hygromycin (Clontech). Single colonies were picked, expanded and screened for desired protein expression in the presence of 1 μg ml^−1^ doxycycline (Clontech).

### Immunoblotting and immunoprecipitation

The Cdc20-pT70 and Cdc20-pS92 antibodies were made in an in-house facility by immunizing rabbits with Cdc20-pT70- or Cdc20-pS92-containing peptides coupled to haemocyanin (Sigma). The purified antibodies were concentrated to 0.8 mg ml^−1^ and used at 1:400 dilution. Antibodies against human Bub1, BubR1, Mad2, Cdc20, Cdc20-pS153, p31^comet^, H2A-pT120 and Ndc80 were described previously^16^,^42^,^53^,^65^,^67^. The following antibodies were purchased from the indicated commercial sources and were used at the indicated dilution: mouse anti-Cdc20 (Santa Cruz; sc-5296; 1:200) and goat anti-Cdc20 (Santa Cruz; sc-1906; 1:200), mouse anti-GAPDH (6C5; Millipore; CB1001; 1:1,000), mouse anti-Myc (9E10; Roche; 11667203001; 1:5,000), mouse anti-tubulin (DM1A; Sigma; T9026; 1:1,000), rabbit anti-H3-pS10 (Millipore; 06-570; 1:1,000), mouse anti-phospho-S/T-P MPM2 (Millipore; 05-368; 1:500) and CREST serum (ImmunoVision; HCT-0100; 1:1,000).

For quantitative immunoblotting, anti-rabbit IgG (H+L) (Dylight 800 conjugates; Cell Signaling; 5151; used at 1:10,000 dilution), anti-mouse IgG (H+L) (Dylight 680 conjugates; Cell Signaling; 5470; used at 1:10,000 dilution) and anti-goat IgG (H+L) IRDye 680RD (LI-COR; 926-68074; used at 1:5,000 dilution) were used as secondary antibodies. The membranes were scanned with the Odyssey Infared Imaging system (LI-COR).

For immunoprecipitation, cell pellets were lysed with the lysis buffer (50 mM Tris-HCl pH 7.7, 120 mM KCl, 0.1% NP-40 and 1 mM dithiothreitol) supplemented with protease inhibitor tablets (Roche), 0.5 μM okadaic acid (LC Labs) and 10 units per ml TurboNuclease (Accelagen). After centrifugation, the supernatants were incubated with antibody-coupled protein A beads (Bio-Rad) for 1–2 h at 4 °C. After being washed, the beads were boiled in SDS sample buffer and analysed by SDS–PAGE and immunoblotting. Uncropped blots are provided in Supplementary Figs 6–8.

### Flow cytometry

Cells were harvested by trypsinization or by shake-off in cases that involved the collection of only mitotic cells. After being washed once with PBS, samples were fixed with cold 70% ethanol. Fixed cells were washed with PBS, permeabilized with 0.25% Triton X-100 (Sigma) in PBS and incubated with the MPM2 antibody (Millipore) diluted in PBS containing 2% BSA. After being washed with PBS containing 2% BSA, cells were incubated with Alexa Fluor 488 donkey anti-mouse secondary antibody (Life Technologies) diluted in PBS containing 2% BSA. After the antibody incubation, cells were washed again with PBS and resuspended in PBS containing 200 μg ml^−1^ DNase-free RNase A (QIAGEN) and 2 μg ml^−1^ propidium iodide (Sigma). Samples were analysed with a FACSCalibur flow cytometer (BD Biosciences) and the data were processed with the FlowJo software (Tree Star).

### Immunofluorescence

Cells were cultured in four-well chamber slides and were transfected with the desired siRNAs. At 20 h after transfection, 2.5 mM thymidine (Sigma) was added for 16 h. Cells were released into fresh medium for another 9 h. Nocodazole (250 nM; Sigma) was added at 2 h before fixation, to depolymerize microtubules and enrich mitotic cells. Cells were washed once with PBS, pre-fixed with ice-cold 0.5% formaldehyde (Sigma) in PBS and pre-extracted with ice-cold 0.2% Triton X-100 (Sigma) in PBS. Cells were then fixed with ice-cold 4% formaldehyde in PBS for 15 min. After being washed with PBS twice, cells were permeabilized with 0.2% Triton X-100 in PBS for another 20 min at room temperature. Cells were stained with mouse anti-Cdc20, human CREST serum and rabbit anti-Bub1 or BubR1 antibodies diluted in PBS containing 3% BSA and 0.2% Triton X-100. After being washed three times with PBS containing 0.2% Triton X-100, cells were incubated with Alexa Fluor 647 goat anti-mouse, Alexa Fluor 568 goat anti-human and Alexa Fluor 488 goat anti-rabbit secondary antibodies (Life Technologies) diluted in PBS containing 3% BSA and 0.2% Triton X-100. After another three washes with PBS containing 0.2% Triton X-100, samples were stained with 1 μg ml^−1^ 4,6-diamidino-2-phenylindole diluted in PBS for 3 min. After a final wash, slides were mounted and analysed with a DeltaVision deconvolution fluorescence microscope (DeltaVision, GE Healthcare).

Images were acquired with a × 100 1.40 numerical aperture UPLS Apochromat N objective (Olympus). A series of *z*-stack images were captured at 0.5-μm intervals. The *z*-stack images were deconvolved using the provided algorithm with the ‘conservative' setting and projected with the ‘sum' method. All images in one experiment were taken with the same light intensity and exposure times. Quantification of the kinetochore signal intensity was performed in ImageJ. A rectangle mask enclosing the CREST signal from a pair of kinetochores was drawn and defined as the region of interest (ROI). The integrated density for the selected ROI was measured in each channel. The normalized intensity was defined as the ratio between the Cdc20 (or Bub1) intensities and the CREST intensities. Ten ROIs per cell were chosen at random and the normalized intensity was calculated for each ROI. The mean value of the ten normalized intensities is used as the normalized intensity of one cell. Twenty-five to 34 cells were quantified for each sample. The graphs and statistics were generated with Prism (GraphPad Software).

### Live-cell imaging

Cells were cultured in four-well chamber coverglass. After transfection of Bub1 siRNA, 2.5 mM thymidine (Sigma) was added for 16 h. Cells were then released into fresh medium for 6 h. Taxol (200 nM; Sigma) was added for another 3 h before the addition of BI 2536 (100 nM; Selleck Chemicals). Live-cell imaging was performed with a DeltaVision microscope (DeltaVision, GE Healthcare). Differential interference contrast images were taken with a × 40 objective (Olympus). For each sample, 15 fields were chosen randomly. Imaging began at about 40 min after BI 2536 was added and lasted for 4 h with images taken at 4-min intervals. Images were processed with ImageJ and the graph was generated with Prism (GraphPad Software).

### Protein expression and purification

Recombinant human Bub1–Bub3, BubR1–Bub3, Mps1 and Mad2 proteins were expressed in and purified from either bacteria or insect cells^68^,^69^. WT or mutant human Strep–His_6_–Bub1 (full-length or Bub1^ΔKinase^ containing residues 1–723) was co-expressed with His_6_–Bub3 in Sf9 cells. Cells were lysed in buffer B (50 mM Tris-HCl pH 7.7, 200 mM NaCl, 1 mM MgCl_2_, 0.3 mM Na_3_VO_4_, 5 mM NaF, 10 mM β-glycerophosphate, 1 mM dithiothreitol and 5% glycerol) supplemented with protease inhibitor tablets (Roche) and 0.5 μM okadaic acid (LC Labs). Strep–His_6_–Bub1–Bub3 complexes were affinity-purified using Strep-Tactin Superflow Plus resin (QIAGEN) and eluted with buffer B supplemented with 15 mM D-desthiobiotin (Sigma). Purified proteins were exchanged into storage buffer II (25 mM Tris-HCl pH 7.4, 200 mM NaCl, 1 mM MgCl_2_, 1.5 mM dithiothreitol and 5% glycerol).

His_6_–BubR1 and His_6_–Bub3 were co-expressed in Sf9 cells. His_6_–Mps1 was also expressed in Sf9 cells. Cells were lysed in the lysis buffer (50 mM Tris-HCl pH 7.7, 150 mM KCl, 0.1% Triton X-100 and 1 mM dithiothreitol) supplemented with protease inhibitor tablets (Roche). After incubation with Ni^2+^-NTA beads (QIAGEN), the beads were washed with the wash buffer (50 mM Tris-HCl pH 7.7, 300 mM KCl, 10 mM imidazole and 1 mM dithiothreitol). Proteins were eluted with elution buffer I (50 mM Tris-HCl pH 7.7, 300 mM KCl, 50 mM imidazole and 1 mM dithiothreitol) followed by elution buffer II (50 mM Tris-HCl pH 7.7, 300 mM KCl, 100 mM imidazole and 1 mM dithiothreitol). The eluates were then exchanged into the storage buffer (50 mM Tris-HCl pH 7.7, 100 mM KCl, 1 mM dithiothreitol and 10% glycerol), aliquoted and flash-frozen in liquid nitrogen.

The coding region of Mad2 protein was cloned into the pQE30 (QIAGEN) with a TEV cleavage site. After expression in bacteria, the His_6_-tagged recombinant protein was purified with Ni^2+^-NTA beads (QIAGEN). The His_6_ tag was then removed using the TEV protease and the Mad2 protein was further purified with Mono-Q and Superdex-75 gel filtration columns (GE Healthcare).

WT and mutant His_6_-tagged human Cdc20^ΔN60^ proteins containing residues 61–499 were purified as previously described^68^ with the following modifications. Sf9 cells expressing various His_6_–Cdc20 proteins were lysed in buffer A (50 mM HEPES pH 6.8, 250 mM KCl, 5% glycerol, 0.1% Triton X-100 and 5 mM β-mercaptoethanol) supplemented with 10 mM imidazole, protease inhibitor tablets (Roche), 0.5 μM okadaic acid (LC Labs) and 10 units per ml TurboNuclease (Accelagen). After incubation, the Cdc20-bound Ni^2+^-NTA beads (QIAGEN) were washed with buffer A containing 20 mM imidazole and then with buffer A containing 50 mM imidazole. Purified proteins were eluted with buffer A containing 250 mM imidazole. After elution, samples were exchanged into storage buffer I (40 mM HEPES pH 6.8, 200 mM KCl, 5% glycerol and 2 mM dithiothreitol) with PD-10 columns (GE Healthcare).

Human GST-Plk1 T210D (a constitutively active mutant) and GST-Cdk1–cyclin B1 kinases were expressed in Sf9 cells. Cells were lysed with buffer B supplemented with protease inhibitor tablets (Sigma). Cleared lysates were incubated with glutathione-Sepharose 4B beads (GE Healthcare). Proteins bound to beads were eluted with buffer B supplemented with 25 mM reduced glutathione (Sigma). Purified Plk1 and Cdk1 were exchanged into storage buffer II and stored at −80 °C.

His_6_-tagged human BubR1 N-terminal fragment (BubR1N) containing residues 1–370 was expressed in bacteria and purified with Ni^2+^-NTA beads (QIAGEN). After the removal of the His_6_ tag by TEV digestion, the BubR1N protein was further purified with Resource Q and Superdex 200 gel filtration columns (GE Healthcare).

### Ubiquitination assays

APC/C was immunopurified from *Xenopus* egg extracts or HeLa cell lysates using the anti-APC3 antibody-coupled protein A beads (Bio-Rad)^68^. The APC/C beads with or without a prior incubation with Cdc20, BubR1, BubR1N, Mad2, Bub1 or Plk1 were then incubated with a ubiquitination reaction mixture. The ubiquitination reaction mixture was prepared with the XB buffer (10 mM HEPES pH 7.7, 100 mM KCl, 0.1 mM CaCl_2_, 1 mM MgCl_2_ and 50 mM sucrose) and contained 1 × energy mixture (containing 7.5 mM phosphocreatine, 1 mM ATP, 100 μM EGTA and 1 mM MgCl_2_), 150 μM bovine ubiquitin (Sigma), 5 μM human Uba1, 2 μM UbcH10 and 5 μM Myc-tagged N-terminal fragment of human cyclin B1 (containing residues 1–97) or 5 μM Myc-tagged securin. Ube2S (a gift from Michael Rape, University of California at Berkeley, Berkeley, CA) was included in certain assays. After incubation at room temperature with gentle shaking for 1 h, the reactions were quenched with 2 × SDS sample buffer. The reaction mixtures were then analysed by SDS–PAGE followed by immunoblotting with mouse anti-Myc antibody (9E10; Roche).

### Kinase and protein-binding assays

For kinase assays, 1 μM Cdc20^ΔN60^ WT or mutants were incubated with different kinases at room temperature for 30 min in 20- μl reactions in the kinase buffer (50 mM Tris-HCl pH 7.7, 100 mM NaCl, 10 mM MgCl_2_, 5 mM NaF, 0.1 mM Na_3_VO_4_, 20 mM β glycerophosphate and 1 mM dithiothreitol) supplemented with 100 μM ATP. Kinases used in this study were as follows: 50 nM Plk1 (with or without 500 nM Bub1^ΔKinase^–Bub3), 40 nM Bub1, 50 nM Cdk1 and 100 nM Mps1. The reaction mixtures were quenched with SDS sample buffer and analysed by SDS–PAGE followed by Coomassie blue staining or immunoblotting.

For protein-binding assays, purified His_6_–Cdc20^ΔN60^ WT or the S92E mutant were immobilized on Ni^2+^-NTA beads (QIAGEN). After being washed twice, the beads were incubated with different combinations of BubR1N and Mad2 proteins for 1 h at room temperature. After being washed five times, bound proteins were eluted with SDS sample buffer and analysed by SDS–PAGE followed by immunoblotting.

## Additional information

**How to cite this article:** Jia, L. *et al*. The Bub1–Plk1 kinase complex promotes spindle checkpoint signalling through Cdc20 phosphorylation. *Nat. Commun.* 7:10818 doi: 10.1038/ncomms10818 (2016).

## Supplementary Material

Supplementary Information Supplementary Figures 1-8
