# Supplementary material for: The Bub1–Plk1 kinase complex promotes spindle checkpoint signalling through Cdc20 phosphorylation
Source: Nat Commun. 2016 Feb 25;7:10818. doi: 10.1038/ncomms10818 (PMC4773433; doi:10.1038/ncomms10818)
Supplement: Supplementary Information — Supplementary Figures 1-8 [file ncomms10818-s1.pdf]

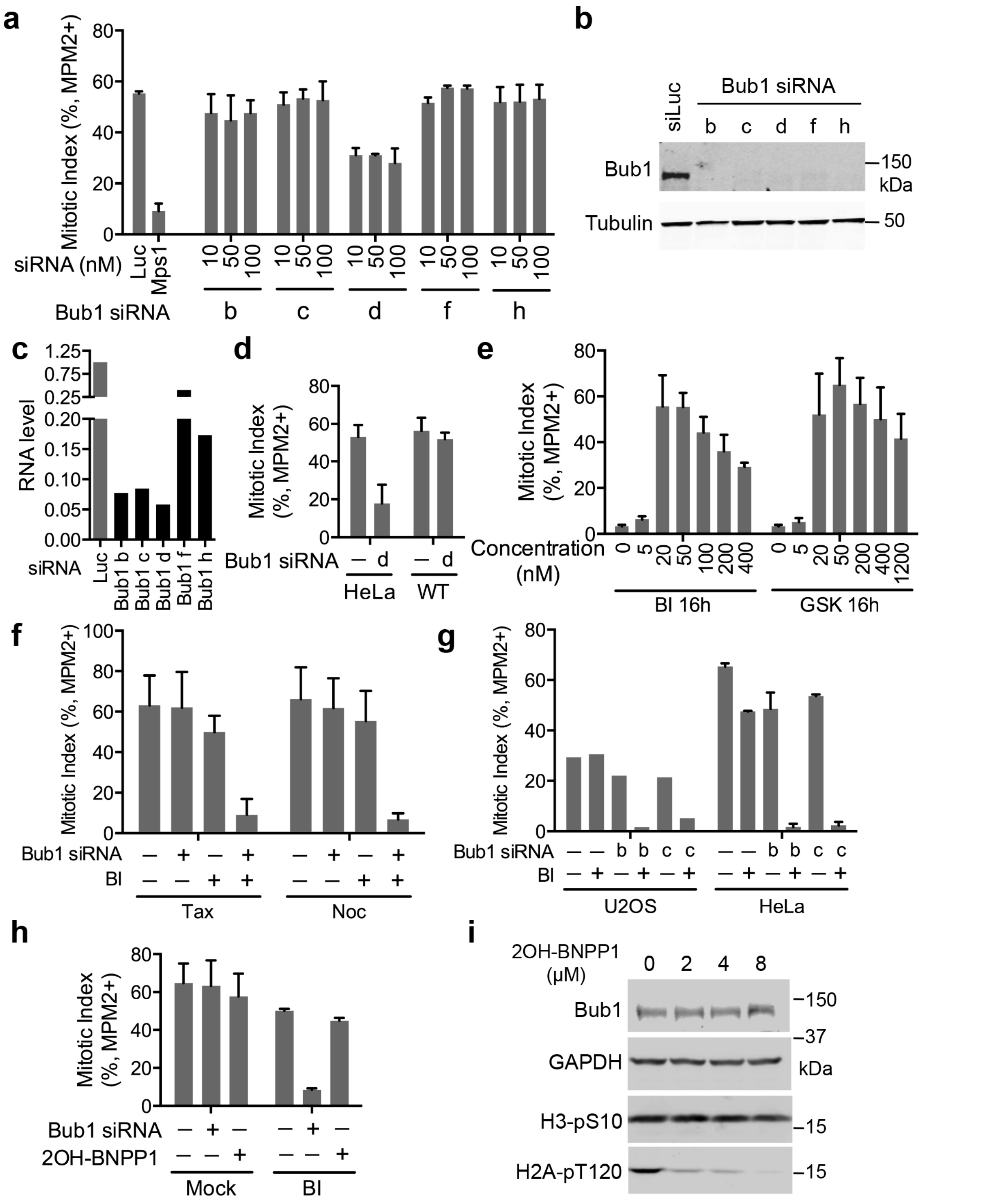

Supplementary Figure 1 | Bub1 depletion and Plk1 inhibition cause strong spindle checkpoint defects. (**a**) Quantification of the mitotic index of HeLa Tet-On cells treated with the indicated siRNAs and 500 nM nocodazole (mean ± range; n = 2 independent experiments). Luc, Luciferase. (**b**) Blots of lysates of HeLa Tet-On cells treated with the indicated siRNAs. (**c**) Quantification of the relative RNA levels in log-phase HeLa Tet-On cells treated with the

indicated siRNAs using quantitative RT-PCR. (**d**) Quantification of the mitotic index of HeLa Tet-On cells and cells stably expressing Bub1 WT treated with 200 nM taxol and the indicated siRNAs (mean ± s.d.; n = 3 independent experiments). (**e**) Quantification of the mitotic index of HeLa Tet-On cells treated with BI 2536 (BI) or GSK461364 (GSK) at the indicated concentrations (mean ± s.d.; n = 3 independent experiments). (**f**) Quantification of the mitotic index of HeLa Tet-On cells treated with or without Bub1 siRNA and BI 2536 (BI) and incubated with 200 nM taxol (Tax) or 500 nM nocodazole (Noc) (mean ± s.d.; n = 3 independent experiments). (**g**) Quantification of the mitotic index of HeLa Tet-On or U2OS cells treated with or without the indicated Bub1 siRNAs and BI 2536 (BI), and incubated with 200 nM taxol (for U2OS cells, n = 1 independent experiment; for HeLa Tet-On cells, error bars show mean ± range; n = 2 independent experiments). (**h**) Quantification of the mitotic index of HeLa Tet-On cells treated with 200 nM taxol in the presence or absence of Bub1 siRNA or inhibitor (2OH-BNPP1) in 200 nM taxol. Mean ± range (n = 2 independent experiments). (**i**) HeLa Tet-On cells were arrested in mitosis with 500 nM nocodazole, and then treated with different doses of the Bub1 inhibitor 2OH-BNPP1. Cell lysates were blotted with the indicated antibodies.

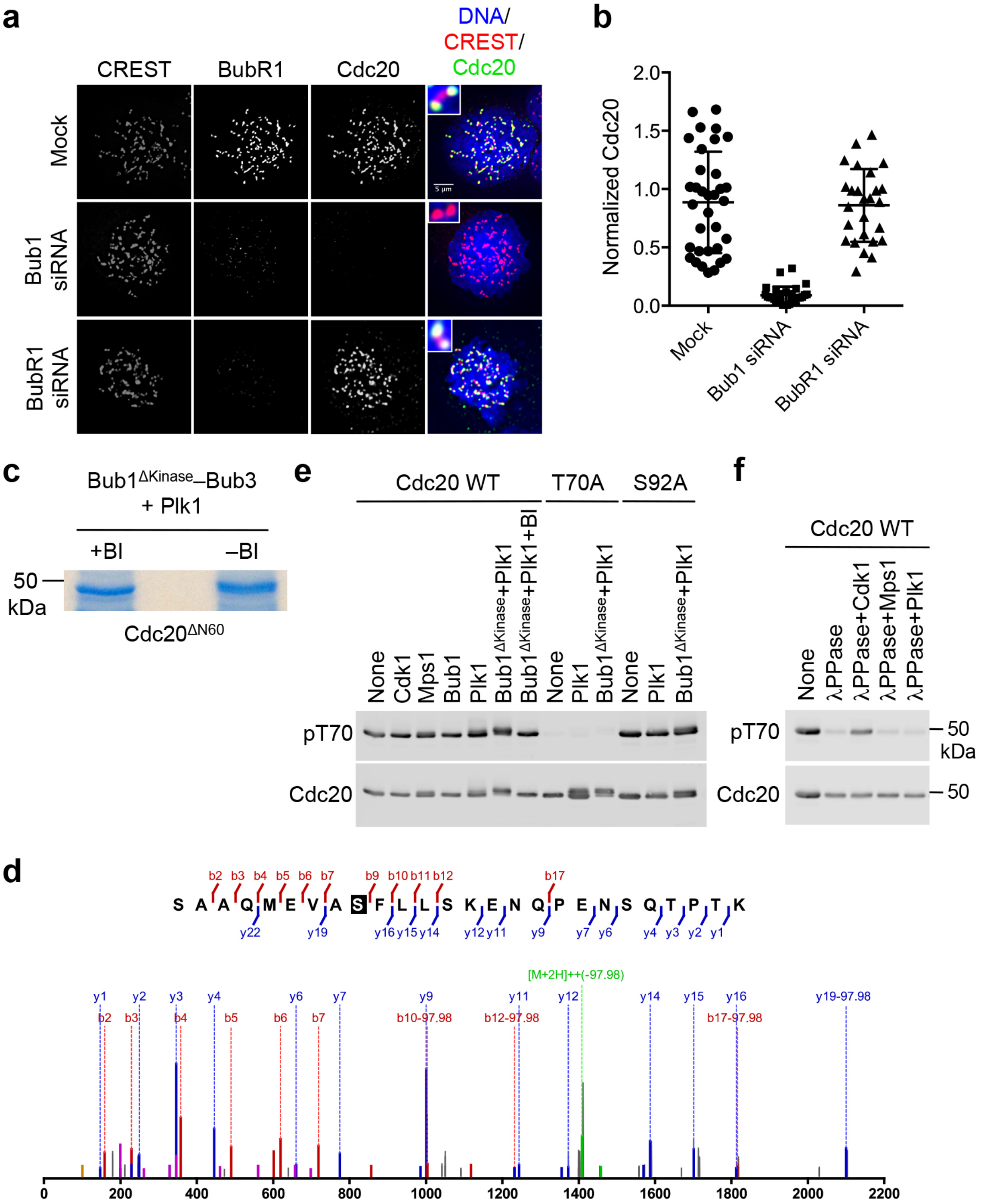

Supplementary Figure 2 | Identification of Cdc20 phosphorylation sites. (**a**) HeLa Tet-On cells were treated with Bub1 or BubR1 siRNAs, and arrested in mitosis with 250 nM nocodazole. Cells were stained with DAPI and the indicated antibodies. Colors of the overlaid channels match those of the label. Selected regions were magnified and shown in insets. Scale bar, 5 µm. (**b**) Quantification of the Cdc20 kinetochore staining intensity in **a** normalized to that of CREST (mean ± s.d.; each dot represents one cell). (**c**) Coomassie blue stained gel of Cdc20$^{\Delta N60}$ treated with Bub1$^{\Delta Kinase}$–Bub3 and Plk1 in the presence or absence of BI 2536. The Cdc20 bands were

excised and analyzed by mass spectrometry. (**d**) Mass spectrum showing the fragmentation pattern of a phospho-S92-containing Cdc20 peptide. (e) Immunoblots of the kinase reactions containing the indicated recombinant kinases and Cdc20 proteins as substrates. BI 2536 (BI) was added to one of these reactions. Cdc20-pT70 and total Cdc20 were blotted. (f) Recombinant Cdc20 WT protein was treated with λ phosphatase (λPPase) before incubation with the indicated kinases. Cdc20-pT70 and total Cdc20 were blotted.

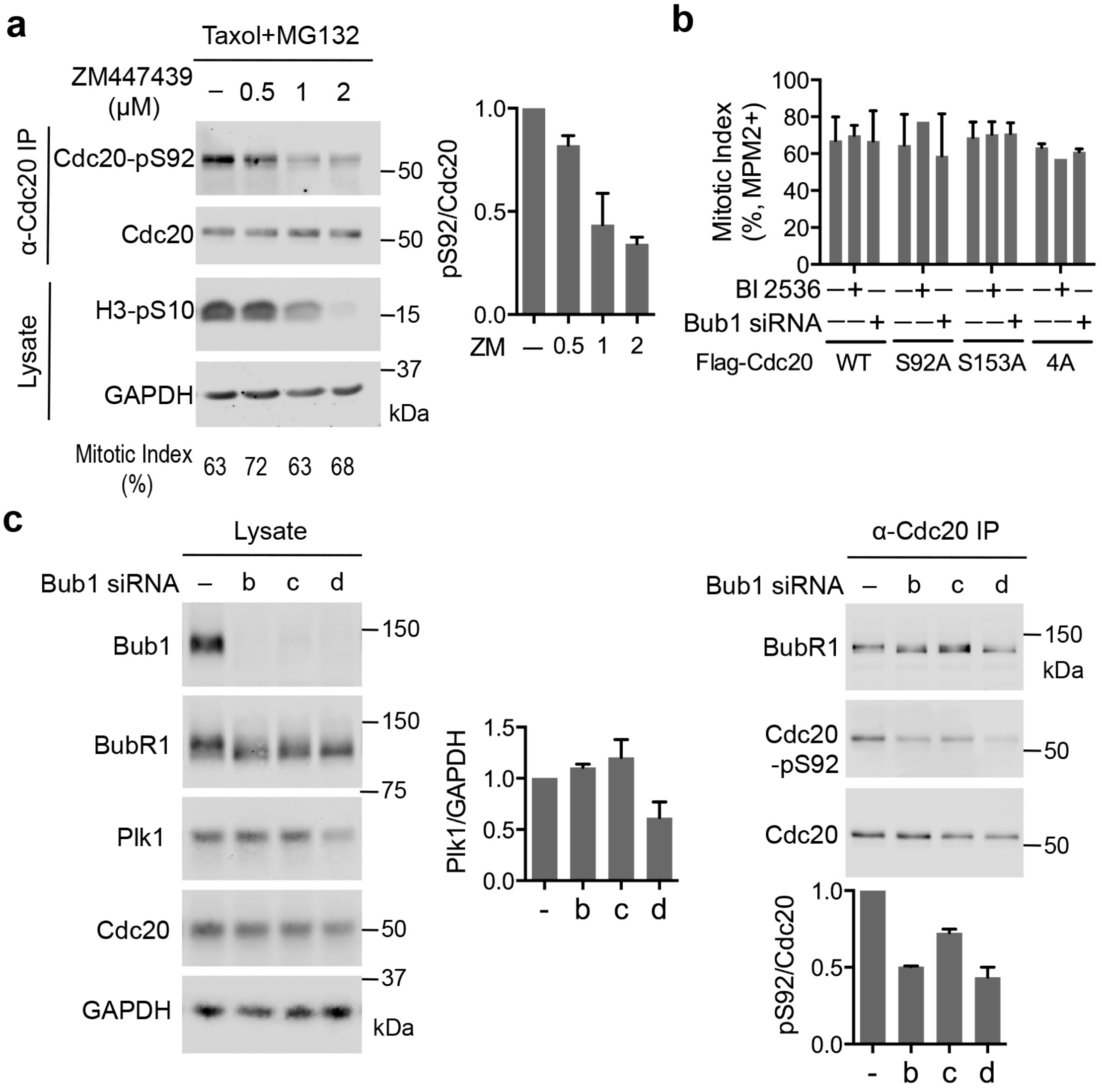

Supplementary Figure 3 | Cdc20 S92 phosphorylation responds to spindle checkpoint signaling. (**a**) HeLa Tet-On cells were arrested in mitosis with 200 nM taxol, then incubated with 10 μM MG132, and different doses of the Aurora B inhibitor ZM447439 for 30 min. The Cdc20 immunoprecipitates (IP) and cell lysates were blotted with the indicated antibodies. The mitotic index of each sample is indicated below each lane. The graph on the right shows the quantification of the Cdc20-pS92 signal normalized to the total Cdc20 level. Mean ± range; n = 2 independent experiments. (**b**) Quantification of the mitotic index of HeLa Tet-On cells stably expressing Flag-Cdc20 WT or mutants with different combinations of BI 2536 and Bub1 siRNA treatments in 500 nM nocodazole. Cdc20 4A, S84A/S92A/S96A/S153A. Mean ± s.d. for columns 1, 2, 3, 4, and 6 (n ≥ 3 independent experiments); mean ± range for columns 7, 8, 9, 10, and 12 (n = 2 independent experiments); one independent experiment for columns 5 and 11. (**c**) Lysates of HeLa Tet-On cells transfected with different Bub1 siRNAs were blotted with the indicated antibodies. Graph on the right shows the quantification of the Plk1 level normalized to that of the loading control GAPDH.

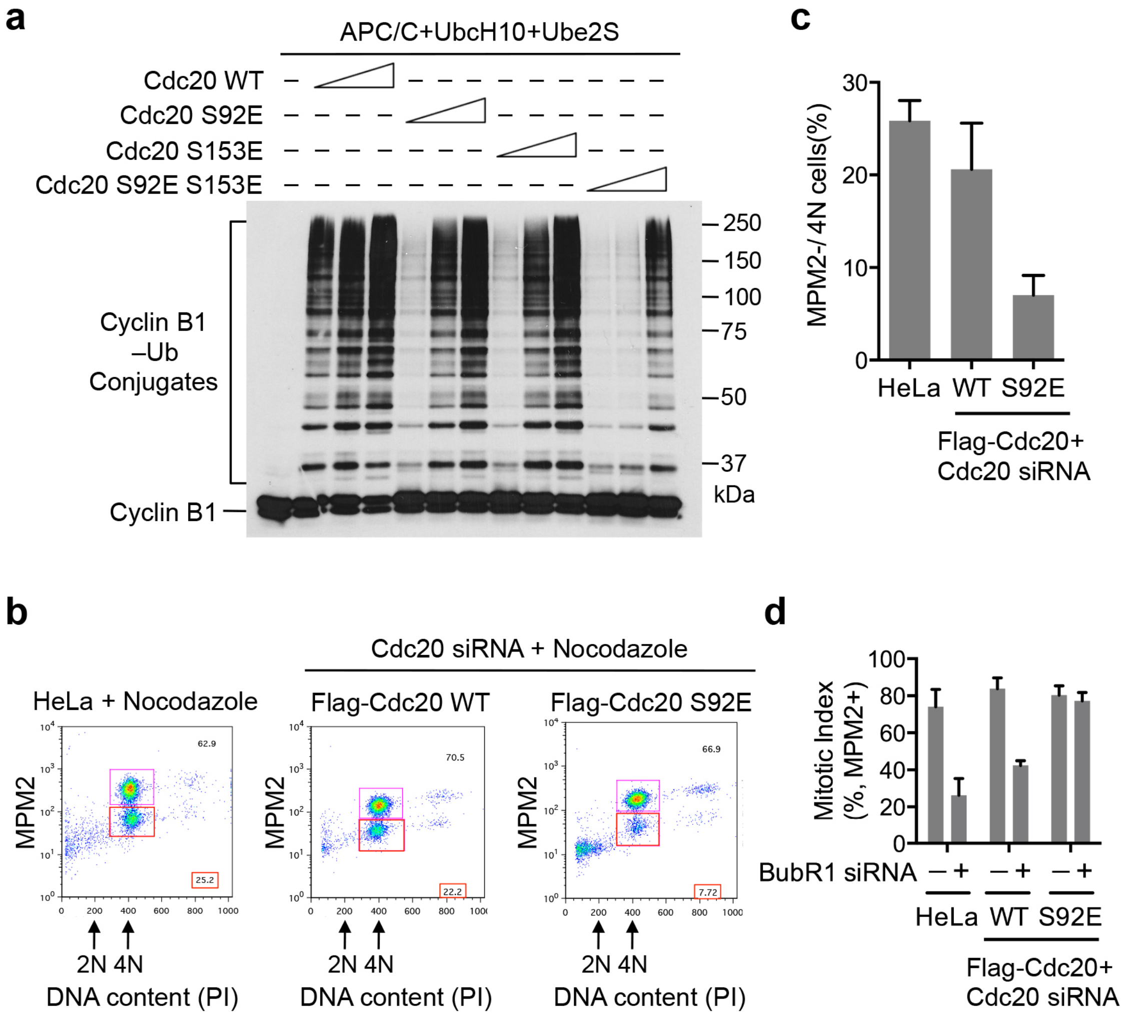

Supplementary Figure 4 | The phospho-mimicking Cdc20 mutants are defective in APC/C activation, suppresses mitotic adaption, and alleviates the requirement for BubR1 in the spindle checkpoint. (**a**) Anti-Myc blot of the APC/C^{Cdc20} *in vitro* ubiquitination reactions using cyclin B1$_{1\text{-}97}$-Myc as the substrate and both UbcH10 and Ube2S as ubiquitin-conjugating enzymes. Different doses of recombinant Cdc20 WT or mutants were incubated with APC/C isolated from *Xenopus* egg extracts. (**b**) Flow cytometry analysis of nocodazole-treated HeLa Tet-On parental cells and cells stably expressing Flag-Cdc20 WT or S92E (with the endogenous Cdc20 depleted). Mitotic cells (defined as MPM2+/4N cells) are indicated by pink boxes. Cells that underwent adaptation and escaped from mitosis (defined as MPM2-/4N cells) are labeled by red boxes, with their percentages indicated. (**c**) Quantification of the percentage of cells in **b** that underwent adaptation (mean ± s.d.; n = 3 independent experiments). (**d**) Quantification of the mitotic index of HeLa Tet-On parental cells and cells stably expressing Flag-Cdc20 WT or S92E treated with the indicated siRNAs and 500 nM nocodazole (mean ± s.d.; n = 3 independent experiments).

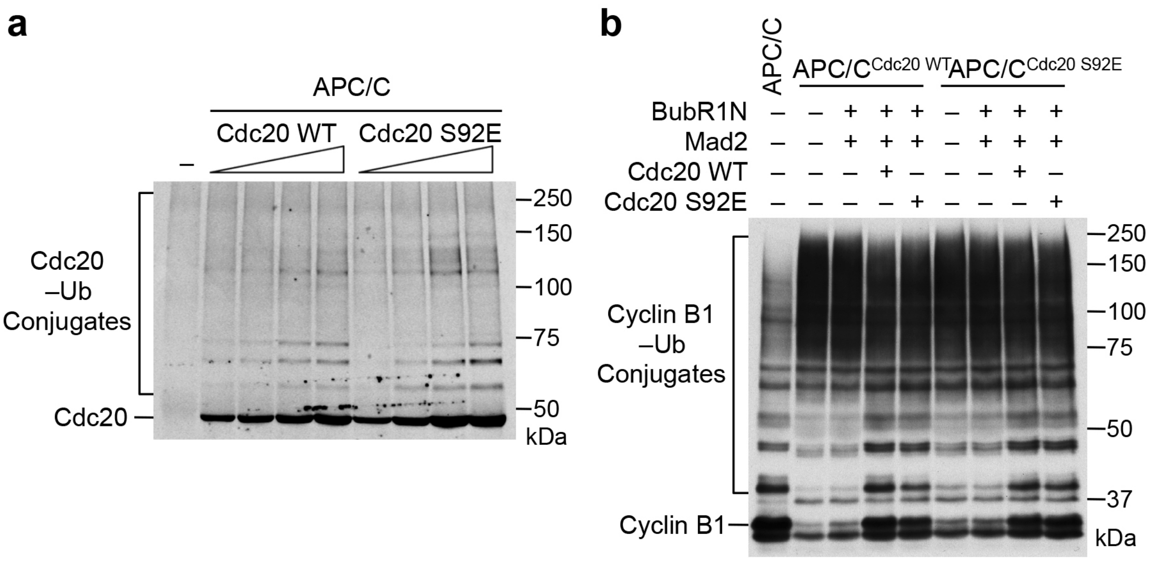

Supplementary Figure 5 | The phospho-mimicking Cdc20 S92E mutation does not affect Cdc20 autoubiquitination or MCC activity. (**a**) Anti-Cdc20 blot of the *in vitro* autoubiquitination reactions of APC/C^{Cdc20} with increasing amounts of recombinant Cdc20 WT or S92E. APC/C was isolated from mitotic HeLa Tet-On cells depleted of endogenous Cdc20. (**b**) Anti-Myc blot of the *in vitro* ubiquitination reactions of the indicated APC/C^{Cdc20} incubated with the indicated proteins and using cyclin B1$_{1\text{-}97}$-Myc as the substrate. See Fig. 6c for experimental design.

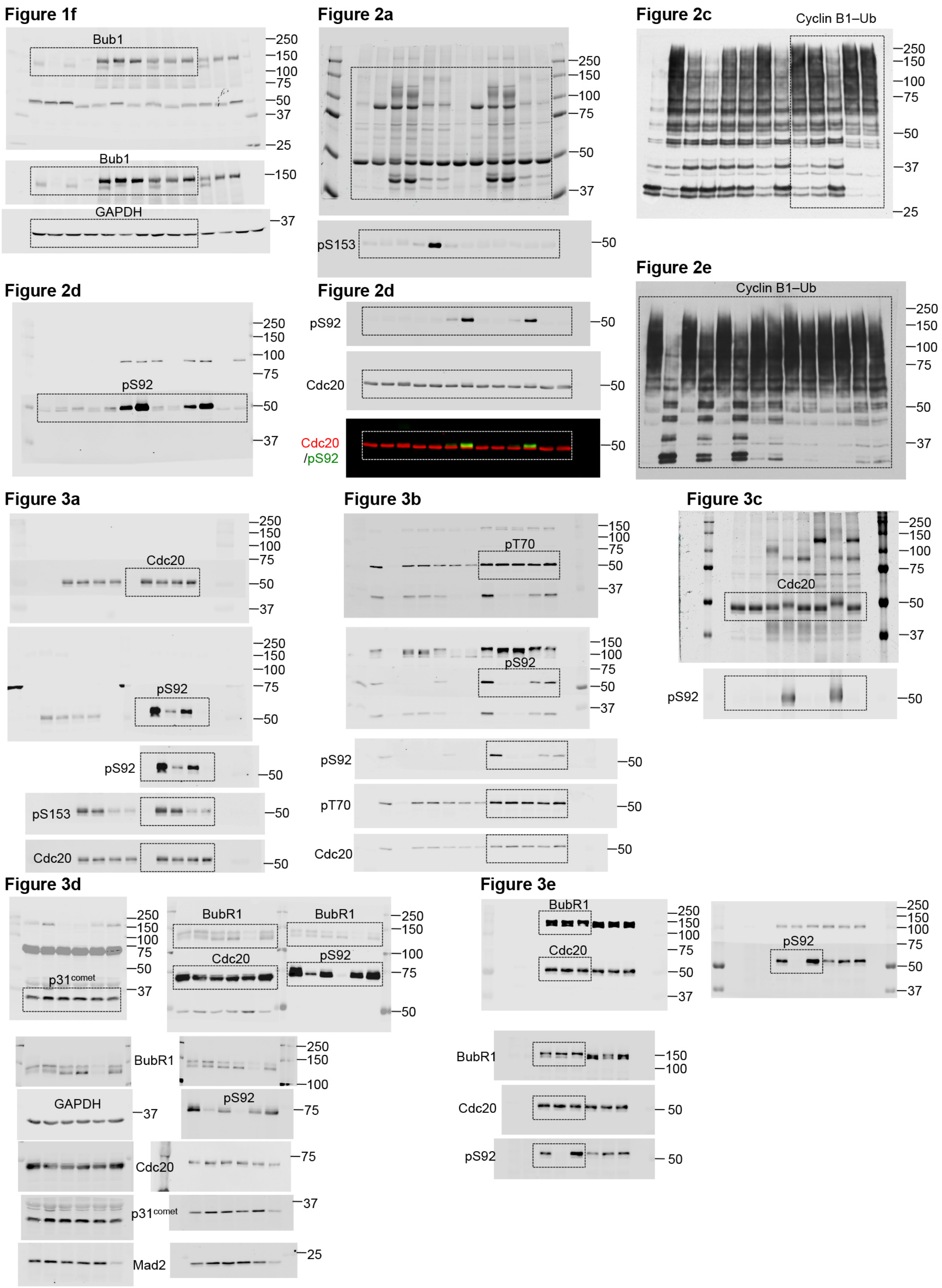

Supplementary Figure 6 | The full uncropped images of blots and gels shown in Figures 1–3.

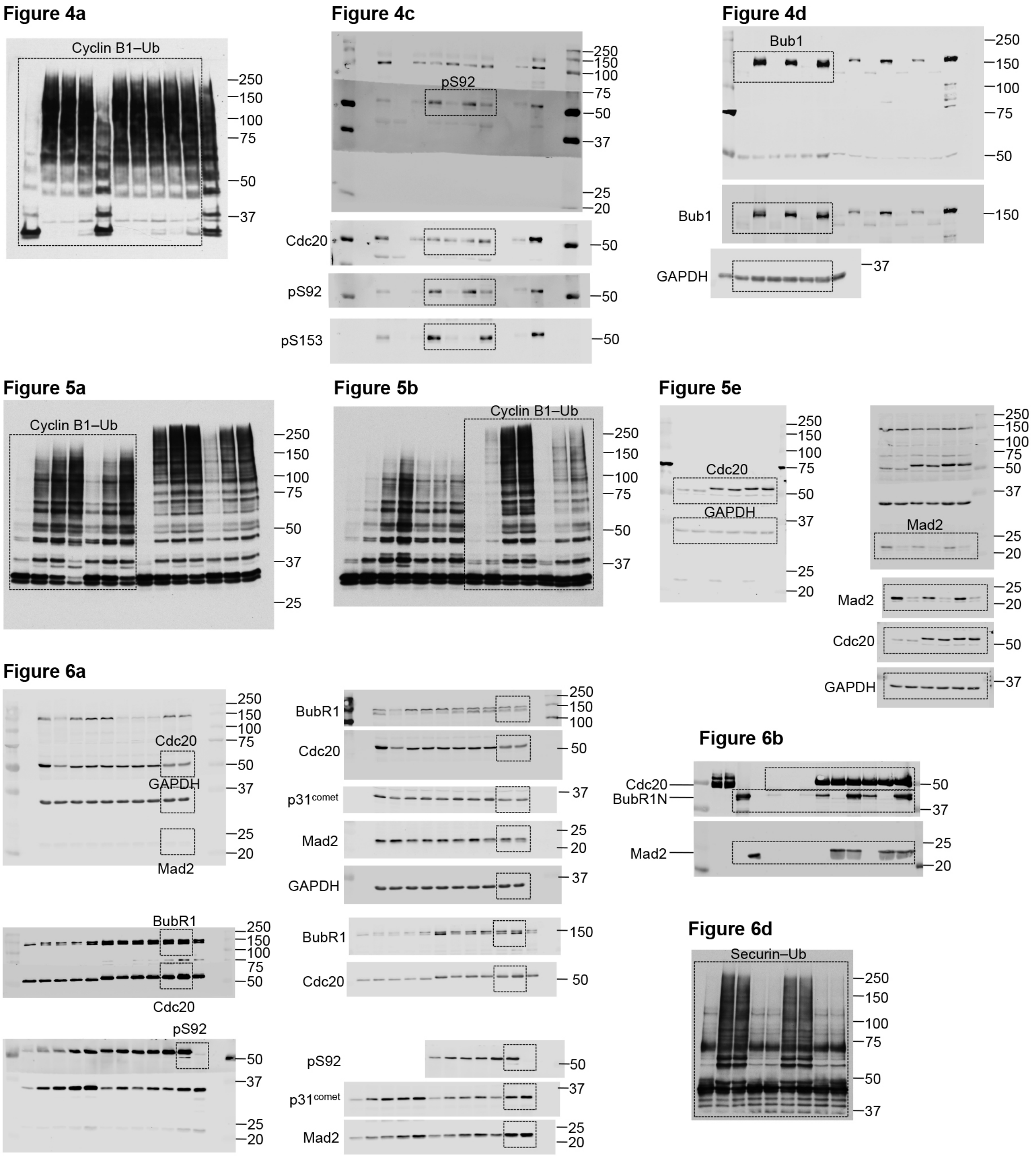

Supplementary Figure 7 | The full uncropped images of blots and gels shown in Figures 4–6.

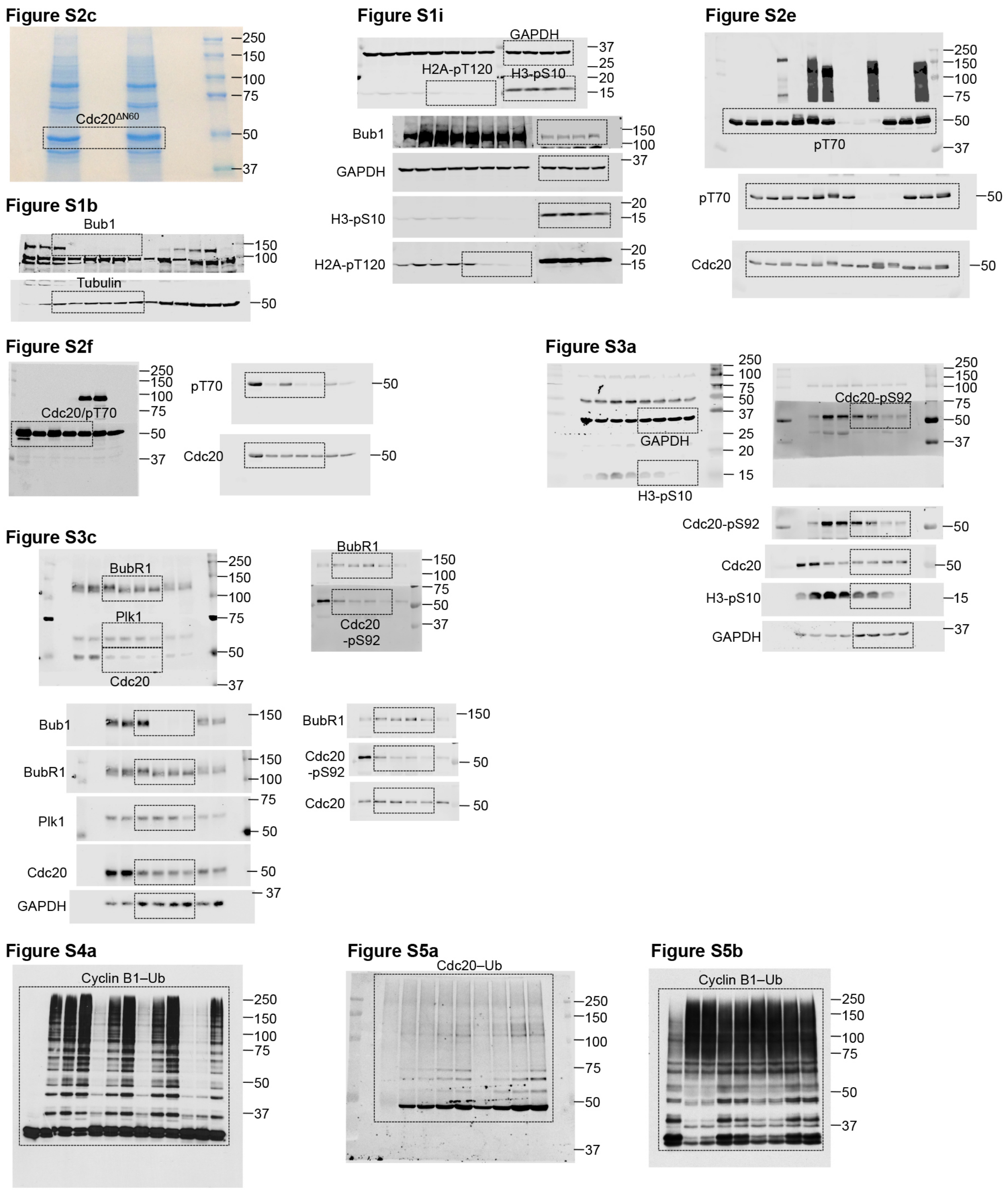

Supplementary Figure 8 | The full uncropped images of blots and gels shown in Supplementary Figures 1–5.
